# Large-scale experimental studies show unexpected amino acid effects on protein expression and solubility *in vivo *in *E. coli*

**DOI:** 10.1186/2042-5783-1-6

**Published:** 2011-06-27

**Authors:** W Nicholson Price, Samuel K Handelman, John K Everett, Saichiu N Tong, Ana Bracic, Jon D Luff, Victor Naumov, Thomas Acton, Philip Manor, Rong Xiao, Burkhard Rost, Gaetano T Montelione, John F Hunt

**Affiliations:** 1 Northeast Structural Genomics Consortium; 2 Department of Biological Sciences, Columbia University, 702A Fairchild Center, MC2434, 1212 Amsterdam Avenue, New York, 10027, USA; 3 Department of Molecular Biology and Biochemistry, Center for Advanced Biotechnology and Medicine, Rutgers University, 679 Hoes Lane, Piscataway, 08854, USA; 4 Wilf Family Department of Politics, New York University, 19 W. 4th Street, New York, 10012, USA; 5 Department of Biochemistry and Molecular Biophysics, Columbia University, 1130 St. Nicholas Avenue, New York, 10032, USA; 6 Department of Biochemistry, Robert Wood Johnson Medical School, University of Medicine and Dentistry of New Jersey, 679 Hoes Lane, Piscataway, 08854, USA

## Abstract

The biochemical and physical factors controlling protein expression level and solubility *in vivo *remain incompletely characterized. To gain insight into the primary sequence features influencing these outcomes, we performed statistical analyses of results from the high-throughput protein-production pipeline of the Northeast Structural Genomics Consortium. Proteins expressed in *E. coli *and consistently purified were scored independently for expression and solubility levels. These parameters nonetheless show a very strong positive correlation. We used logistic regressions to determine whether they are systematically influenced by fractional amino acid composition or several bulk sequence parameters including hydrophobicity, sidechain entropy, electrostatic charge, and predicted backbone disorder. Decreasing hydrophobicity correlates with higher expression and solubility levels, but this correlation apparently derives solely from the beneficial effect of three charged amino acids, at least for bacterial proteins. In fact, the three most hydrophobic residues showed very different correlations with solubility level. Leu showed the strongest negative correlation among amino acids, while Ile showed a slightly positive correlation in most data segments. Several other amino acids also had unexpected effects. Notably, Arg correlated with decreased expression and, most surprisingly, solubility of bacterial proteins, an effect only partially attributable to rare codons. However, rare codons did significantly reduce expression despite use of a codon-enhanced strain. Additional analyses suggest that positively but not negatively charged amino acids may reduce translation efficiency in *E. coli *irrespective of codon usage. While some observed effects may reflect indirect evolutionary correlations, others may reflect basic physicochemical phenomena. We used these results to construct and validate predictors of expression and solubility levels and overall protein usability, and we propose new strategies to be explored for engineering improved protein expression and solubility.

## Background

Overexpression of recombinant proteins is a central method in contemporary biochemistry, structural biology, and biotechnology. Unfortunately, it is often a significant experimental challenge [1,2]. Many recombinant proteins express at low levels or not at all, and those proteins which express at high levels but in an insoluble form cannot be used without applying technically challenging refolding procedures [3]. Despite significant experimental [1,2,4-7] and computational [8-12] progress, the physical mechanisms underlying this variability remain poorly understood. The goal of the work reported in this paper is to increase understanding of the sequence parameters and physicochemical mechanisms influencing protein overexpression and solubility *in vivo.*

Most existing experimental techniques to improve yield of soluble protein focus on optimization of factors extrinsic to the target protein itself. Experimental techniques for increasing expression have been reviewed elsewhere [1,2]; they include co-expression of fusion partners (including MBP [13] and smt [14] ), codon enhancement [15], or optimization [16,17] (including removal of 5' RNA secondary structure [18]), and the use of protease-deficient strains [19]. Techniques that have been developed specifically to improve solubility of recombinant proteins include chaperone co-expression [3], fusion to solubility-enhancing tags or protein domains [13,4], heat shock [20] or expression at lower temperature [1], expression in a different growth medium [1], reduction of protein expression level (*e.g.*, by using less inducer or a weaker promoter [21]), directed evolution [22,23], and rational mutagenesis [24]. Of these, only the last relies on understanding the properties of the protein itself, rather than on modifying an external factor. Intrinsic biophysical features influencing protein expression and solubility *in vivo *have received comparatively little systematic study, at least in part because of the complexity of the physicochemical processes determining outcome.

Net protein expression level *in vivo *reflects the balance of a series of different biochemical processes, including gene transcription rate, mRNA stability, mRNA translation rate into protein, and protein degradation rate. Different amino acids could potentially influence all of these steps, in some cases directly and in others indirectly. Important indirect effects include amino-acid influence on the local and global conformational stability of the protein, which can impact degradation rate [25], and codon usage, which can impact protein translation efficiency [26] as well as mRNA transcription rate via the GC content of the gene [27]. *In vivo *solubility level is similarly influenced by a number of different physicochemical factors, including the folding efficiency [7,17,23] and thermodynamic stability [6,25,28,29] of the protein, its tendency to form non-native aggregates including amyloid structures [30-32], and its inherent thermodynamic solubility (*i.e.*, the chemical potential of the dissolved state compared to native-state aggregates including crystalline phases and amorphous precipitates) [6,28,29,33]. Different amino acids could also potentially influence all of these physical processes contributing to the recovery of protein in soluble form after expression *in vivo*. Indirect evolutionary effects, in which two different physicochemical properties are simultaneously selected via the same evolutionary process, represent an additional complication in extracting mechanistic inferences from correlations in a large-scale biological dataset. Therefore, the influence of bulk amino-acid properties on protein expression and solubility *in vivo *in a large-scale experimental study could have many possible physicochemical explanations, which would require critical evaluation via other experimental approaches. Nonetheless, due to the absence of conceptual bias, datamining large-scale *in vivo *expression results offers a unique opportunity to detect biologically significant effects that have evaded characterization via hypothesis-driven experimentation [34].

Many previous studies have focused on understanding sequence correlations with specific physicochemical properties of proteins. While a previous study of purified NESG proteins indicated no correlation between mean hydrophobicity and folding stability of a set of predominantly mesophilic eubacterial proteins [35], studies of sequence bias in hyperthermophilic proteins suggest an enhanced content of both hydrophobic and charged amino acids in hyperstable proteins at the expense of polar amino acids [36,37], with the charged amino acids tending to participate in cooperative salt-bridging networks [37-40]. Analysis of the predicted proteomes from the genomes of a large set of bacteria show that the fractional content of a specific set of residues (IVYWREL) is a good predictor of the optimal growth rate of the organism, which was interpreted to reflect the influence of these amino acids in enhancing protein stability [27]. However, this mechanistic inference was not tested experimentally, so other explanations for the observed correlations have not been excluded (*e.g.*, explanations related to metabolic constraints on hyperthermophiles or the altered thermodynamic properties of water at elevated temperatures).

Other studies have examined sequence correlations with protein solubility using widely varying experimental approaches and solubility assays. One study examined the yield of soluble protein after expressing all *E. coli *proteins individually in a cell-free, *in vitro *translation system [34]. A bimodal distribution was observed, with higher soluble yield being positively correlated with lower isoelectric point and the prevalence of negatively charged amino acids but not correlated with the prevalence of hydrophobic amino acids. Another study examined the influence of the 20 amino acids at a single surface-exposed site on the thermodynamic solubility of RNase Sa at high ammonium sulfate concentration [6]. Asp, Glu, and Ser were observed to have a more favorable influence than other hydrophilic amino acids at this position [6]. Finally, an analysis of cytosolic proteins in *E. coli *showed a positive correlation between mRNA abundance and the *in vitro *solubility of the corresponding protein, both of which were positively correlated with several parameters including hydrophilicity, low GC content, and α-helical content [30,31]. This analysis, however, was based on a small set of proteins forming amyloid-like insoluble structures, and the observed correlations could be influenced by the specific stereochemistry of the ordered aggregates formed by these proteins.

One widely applied approach to improving expression and solubility is to avoid difficult proteins in favor of homologous targets [41]. Therefore, the ability to identify primary sequence factors that impact these factors is technologically valuable. Previous predictive work has studied the effect of codon usage [5] and RNA secondary structure [5,42] on protein expression. Some predictive analyses of solubility have examined a small set of global sequence characteristics based on published datasets of relatively small size [8,9]. Others have used proxy criteria from larger datasets rather than direct experimental observations [10,11,43] (*e.g.*, not progressing past the "expressed" stage in TargetDB [44], which tracks the progress of structural genomics projects). These analyses have employed sophisticated computational methods that can provide reasonable predictors of outcome but that make it difficult to extract qualitative trends providing conceptual insight into the sequence determinants of protein expression and solubility.

Despite the many valuable insights and tools that have emerged from these previous studies, substantial uncertainty remains concerning the biochemical and physical factors that influence protein expression and solubility level *in vivo*. To deepen understanding of these factors, we analyzed results from the protein production pipeline of the NESG, where over 19,000 protein constructs have been taken through the same cloning and *E. coli *expression pipeline [45]. These protein constructs were scored independently for their expression level and the fraction of the expressed protein recovered in soluble form. Uniform processing of thousands of protein expression experiments should remove methodological variances that could impact outcome, as well as stochastic variations in individual experiments [46], enabling significant trends to be observed more clearly. Earlier studies of the Northeast Structural Genomics Consortium (NESG - http://www.nesg.org) pipeline have elucidated some determinants of experimental performance [43,35]. Our statistical analyses of a much larger number of observations from this high-throughput experimental pipeline provide new insight into the amino acid sequence properties that influence protein expression and solubility level *in vivo *and reveal a number of surprising sequence correlations that have evaded characterization via traditional mechanistic experimentation. Some of these correlations are likely to reflect the biochemical and physical properties of the individual amino acids, suggesting new approaches to be evaluated for their efficacy in engineering improved protein expression results.

## Results

### Characteristics of the high-throughput protein-expression dataset

Comprehensive statistical analyses were performed on results obtained from a set of 9,644 target proteins taken through the uniform protein expression and purification pipeline of the NESG between 2001 and mid-2008 (82% eubacterial, 12% archaebacterial, 6% human, and 0.3% from other eukaryotes). A single construct was evaluated for each. Some targets were individual domains taken from multi-domain proteins. Targets with large low-complexity regions, predicted transmembrane α-helices, or predicted signal peptides were excluded from this set, although a small fraction are predicted to contain the latter features by some analysis methods (less than 8% of the total - see Methods). Proteins carrying short hexa-histidine tags were expressed overnight at 18°C in *E. coli *BL21λ(DE3) cells from a T7-polymerase-based pET vector [45]. The expression strains also harbored the pMGK plasmid overespressing three tRNAs cognate to rare codons for Arg and Ile residues [45]. A subset of 7,733 of these proteins (designated the "Analysis Dataset") was used for model development and initial regressions, while the remaining 1,911 proteins (designated the "Test Dataset") were set aside for use solely in model validation.

Based on small-scale expression trials conducted on at least two clones, each construct was assigned integer scores from 0 to 5 independently for its expression level (E), based on the total amount of protein observed on a Coomassie-Blue-stained SDS-PAGE gel, and for the proportion of the expressed protein recovered in the soluble fraction (solubility score or S), based on its yield in the supernatant after low-speed centrifugation to remove insoluble material. An S value of 0 indicates no recovery in the supernatant, while a value of 5 indicates ~100% recovery; intermediate values are roughly linearly proportional to the level of yield between these two extremes. S values were not recorded for proteins with E = 0. The solubility score S reflects a combination of physical factors including *in vivo *folding efficiency [47,48], thermodynamic stability [6,23,49], and aggregation propensity [30,31,50] in addition to the thermodynamic solubility of the natively folded protein. Nonetheless, it provides both a practical measure of success in producing useful protein preparations and a tool for generating hypotheses concerning physicochemical factors influencing soluble protein expression *in vivo *in *E. coli*. Our explicit experimental observations provide more detail than datasets previously used to develop predictors of protein solubility [8,10,11], which have segregated proteins based on binary criteria (*e.g.*, the absence or presence of inclusion bodies).

Some data analyses were performed based on the value of E*S, *i.e.*, the product of the expression level and solubility score. During the relevant time period, the operational requirement for protein scale-up for purification by the NESG was having E*S > 11. Therefore, an E*S product above this threshold represents a criterion for practical utility or "usability."

Experiments on multiple clones for each construct show equivalently good reproducibility of both E and S values in our large-scale experimental dataset (Additional file 1, Figure S1A). Furthermore, the E and S values observed in subsequent large-scale expression/purification procedures closely parallels those observed in small-scale expression trials on the same clone (Additional file 1, Figure S1B). These observations indicate that our large-scale experimental dataset is not compromised by problems related to stochastic variation in expression [46].

For the ~25% of protein constructs for which different E or S values were observed for different clones, the maximum observed values were used for statistical analyses of the primary dataset examined in this paper. This approach was motivated by the expectation that most experimental errors in the protein-production pipeline should lower E or S value. It is justified by analyses of alternative datasets employing a series of different approaches to deal with discrepancies in observed E and S values, as presented in more detail below.

Additional file 1, Figure S2 shows that there is little correlation between E or S and thermal stability (T_m_) for 75 purified bacterial proteins randomly selected from our expression dataset. While this analysis was limited to proteins with E*S > 11 (because proteins falling below this threshold were not purified), it suggests that thermal stability is not a major determinant of expression level or solubility score for proteins expressed in soluble form at a moderate or high level.

### Strong correlation between expression level and solubility score

**Figure 1:**
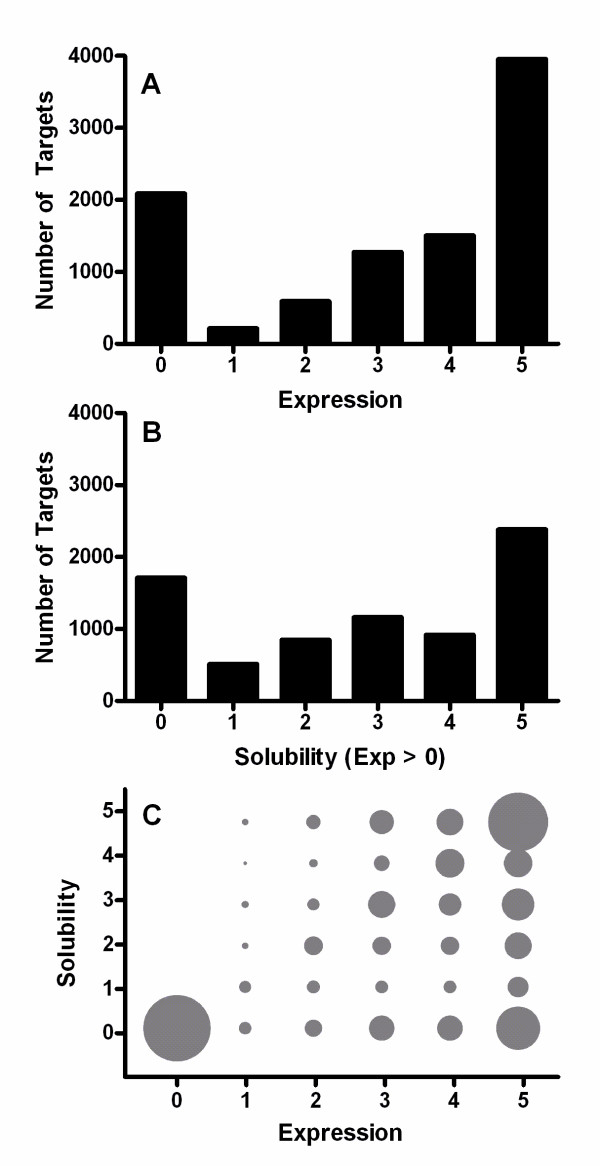
**Distribution of proteins by expression level and solubility score**. The principal dataset analyzed in this paper contains 9,644 target proteins that went through small-scale *E. coli *expression trials in the NESG protein-production pipeline. It was randomly divided into an Analysis Dataset used for regression analyses and model development (7,733 proteins) and a Test Dataset used for model validation (the remaining 1,911 proteins). Each protein was assigned independent integer values from 0-5 for its expression level (E) and solubility score (S), as assessed by visual inspection of a Coomasie-Blue-stained SDS-PAGE gel containing the total cell extract and corresponding soluble fraction. **(A) **Distribution of E scores in the combined Analysis and Test Datasets. **(B) **Distribution of S scores for proteins with non-zero expression in the combined Datasets. **(C) **Bubble-plot showing the relative number of proteins in bins segregated simultaneously by both expression level and solubility score. The area of each bubble is proportional to the number of proteins with that exact combination of E and S values. Therefore, each column in the plot represents all proteins with the corresponding E score, while each row represents all proteins with the corresponding S score. For example, the upper-right-most bubble shows that 1,653 constructs had a S score of 5 among the 3,957 proteins with an E score of 5, which are represented by the total area of all bubbles in the right-most column. In this dataset, 3,880 proteins were considered useable for purification and biophysical characterization, as defined by having E*S > 11.

Although all combinations of E and S values were observed in our large-scale dataset, the majority of proteins scored at the extremes of both score ranges (roughly 65% in each case) (Figure 1). Higher E correlates very strongly with higher S in this dataset. Excluding proteins showing no expression, E predicted S more significantly (p = 4.5 × 10^-67^) than any of the sequence parameters evaluated below. These results are consistent with those obtained from high-throughput experiments examining expression of all *E. coli *proteins in an *E. coli *cell-free *in vitro *translation system [34]. A reanalysis comparing the solubility levels observed in this study to the *in vivo *expression levels assessed using quantitative mass spectrometry [51] also shows an equivalent trend with higher expression *in vivo *correlating with higher soluble yield *in vitro *(G. Boel, L. Brown, and J.F. Hunt, unpublished results).

The strong positive correlation between E and S complicates efforts to interpret the influence of sequence parameters on expression level *vs*. solubility score when the parameters have a consistent effect on both. However, parameters showing a substantially stronger effect on one of the two scores are more likely to act mechanistically on one of the related biochemical processes (*i.e.*, translation/degradation rate *vs*. solubility/aggregation), while parameters showing opposite effects on the two scores can be interpreted to have opposing effects on these processes.

### Logistic regression to evaluate dependence on primary sequence parameters

Because E and S values are non-continuous, ordinary least-squares regressions are not appropriate to evaluate their relationship to sequence parameters. Therefore, we used logistic regressions to determine which sequence parameters significantly predict expression level, solubility score, or usability. Logistic regression determines whether there is a significant relationship between a continuous independent variable (*e.g.*, fractional amino acid content) and a categorical output variable [52]. The method involves calculating the odds-ratio for the different outcomes for defined ranges of the continuous variable and then performing a linear regression against the logarithm of the odds-ratio [52,53]. As opposed to a standard logistic regression, which applies this analysis to a single binary outcome, an ordinal logistic regression applies a similar analysis to the probability of being at or below each successive value of the categorical output variable (*e.g.*, E or S) [52]. The sequence parameters (continuous independent variables) that we analyzed using ordinal logistic regression included the fractional content of each amino acid and twelve aggregate parameters, including protein length, GRAVY (the GRand AVerage of hydropathY [54]), mean side chain entropy (SCE) for all residues and those predicted to be surface-exposed by PHD/PROF, isoelectric point, and six additional electrostatic charge variables (Table 1).

**Table 1:** Sequence parameter names and formulae^†^

Variable Name	Parameter	Parameter Formula
***x ***(*e.g*., a, c)	Fractional content of residue *x*	(count of residue *x*)/(chain length)

***x*b **(*e.g*., cb, db)	predicted buried amino acid fraction	(number of residue *x *predicted buried by PHD/PROF)/(chain length)

***x*e **(*e.g*., de, ee)	predicted exposed amino acid fraction	(number of residue *x *predicted exposed by PHD/PROF)/(chain length)

**gravy**	GRAVY/hydrophobicity	mean residue hydrophobicity

**sce**	sidechain entropy	mean sidechain entropy of all residues

**esce**	predicted exposed sidechain entropy	mean sidechain entropy of residues predicted exposed by PHD/PROF

**numcharge**	number of charged residues	R + K + D + E

**netcharge**	net charge	R + K - D - E

**absnetcharge**	absolute net charge	|R + K - D - E|

**fracnumcharge**	fraction of charged residues	(R + D + D + E)/chain length

**fracnetcharge**	fractional net charge	(R + K - D - E)/(chain length)

**fracabsnetcharge**	fractional absolute net charge	|R + K - D - E|/(chain length)

**diso**	fraction predicted disordered residues	(number of amino acids predicted disordered by DISOPRED2)/(chain length)

**length**	chain length	number of residues

**pi **	isoelectric point	ExPASy pI (http://ca.expasy.org/tools/pi_tool.html)

^† ^Sequence parameters analyzed for correlation with expression, solubility, and usability. Sixty amino acid variables were considered, including the fractional content of each amino acid as well as this content divided into separate parameters for residues predicted by the program PHD/PROF to be solvent-exposed *vs*. buried. Twelve compound variables were also considered, including hydrophobicity (GRAVY) [32], mean side-chain entropy (SCE) [39] for all residues or only those predicted by PHD/PROF [61,80,81] to be surface-exposed, several electrostatic charge variables, the fraction of residues predicted by the program DISOPRED2 [78] to be disordered, isoelectric point, and construct chain length.

**Figure 2:**
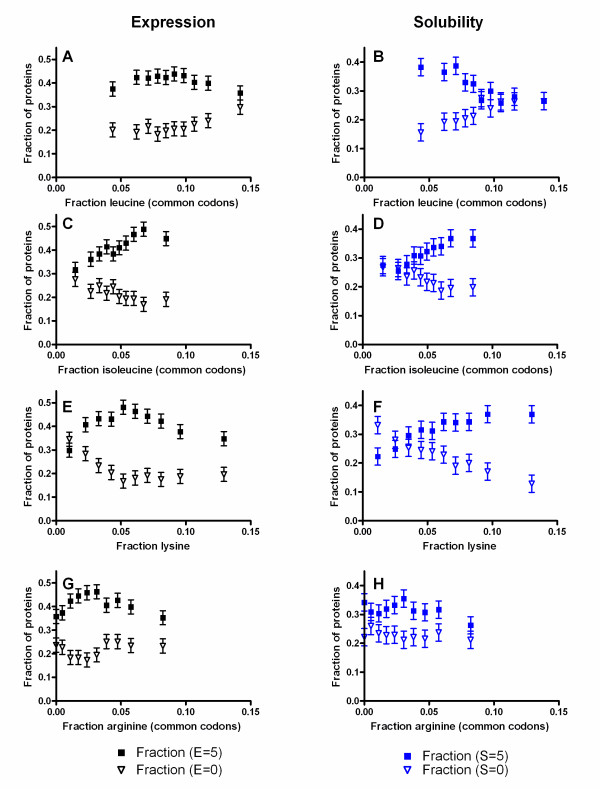
**Influence of four amino acids on expression level and solubility score**. Proteins in the Analysis Dataset presented in Figure 1 were divided into 10 bins based on their fractional content of a single amino acid. The plots show the fractions of proteins within each bin scoring either 0 (no expression or completely insoluble) or 5 (very high expression or solubility); the error bars represent 95% confidence intervals based on counting statistics. Plots are shown for lysine (panels **A **and **B**), arginine (panels **C **and **D**), leucine (panels **E **and **F**), and isoleucine (panels **G **and **H**). The analyses presented in these data panels, but none of the others in the main text, were performed exclusively on the fraction of each amino encoded by common codons in order to factor-out the influence of rare codons (which is explained below).

Figure 2 shows the relationship between two such sequence parameters and the probability of a protein construct exhibiting the lowest (0) or highest (5) E or S values, while Additional file 1, Figure S3 shows the full E and S score distributions for three different sequence parameters exhibiting significant correlations. Lys and Arg would be expected (based on physicochemical similarity) to have equivalent effects on both E and S, as would Leu and Ile. However, the fractional content of Lys correlates positively with S, while the fractional content of Arg shows a small negative correlation with S. Moreover, the fractional content of Ile shows positive correlations with both E and S, while the fractional content of Leu has little impact on E but shows a strong negative correlation with S. (Note that the analyses presented in Figure 2 were limited to the fraction of each amino acid encoded by common codons to exclude any potential influence from rare codons, as addressed in more depth below; however, most graphs and analyses presented in this paper include all synonymous codons for any given amino acid.)

**Figure 3:**
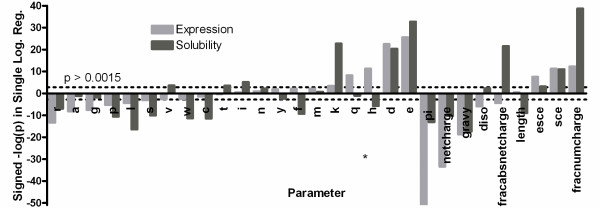
**Statistical significance of primary sequence parameters in predicting outcome**. Ordinal logistic regressions were performed to evaluate whether sequence parameters have a significant influence on the expression levels and solubility scores observed for proteins in the Analysis Dataset (Figure 1). Calculations based on E employed a training set of 7,733 proteins, while those based on S employed the 6,046 proteins in this set with E >0. The ordinate, labeled "Signed -log(p)", shows the negative of the base-10 logarithm of the p-value for the corresponding regression multiplied by the sign of that regression's slope. This scales monotonically with the parameter's "predictive value" (the product of the parameter's regression slope and standard deviation in the dataset). Parameters are arranged by the strength of their influence on E value after segregation of fractional amino acid content from compound sequence parameters. The dotted line shows a Bonferroni-corrected significance threshold of 0.0015. The naïvely counterintuitive negative correlations between net electrostatic charge and both E and S derive from two reinforcing sources. Negatively charged residues have a beneficial/positive influence on both E and S (Additional file 1, Figure S4), which makes the regression slopes negative due to the negative mathematical value of their charge. In the case of E, this effect is reinforced by the deleterious influence of positively charged residues, which makes the regression slope negative for this mathematically positive parameter. The deleterious influence of isoelectric point (pI) on E and S, which has been noted previously [82], is attributable to similar causes (Figure 1 & Additional file 1, Figure S4).

Ordinal logistic regressions were used to assess the statistical significance of the relationship between each of the primary sequence parameters enumerated above and the experimental outcomes observed in our large-scale dataset. Figure 3 shows that many of these sequence parameters have a significant influence on outcome. The values plotted in Figure 3 are the negative of the base-10 logarithm of the p-value for the ordinal logistic regression multiplied by the sign of the slope of this regression (*i.e.*, ±1), so positive correlations yield positive values and negative correlations yield negative values on this graph. These values scale monotonically with the "predictive value" of the parameter, which is defined as the product of its regression slope (which measures the magnitude of its influence) and its standard deviation (which normalizes for its range in the dataset).

**Figure 4:**
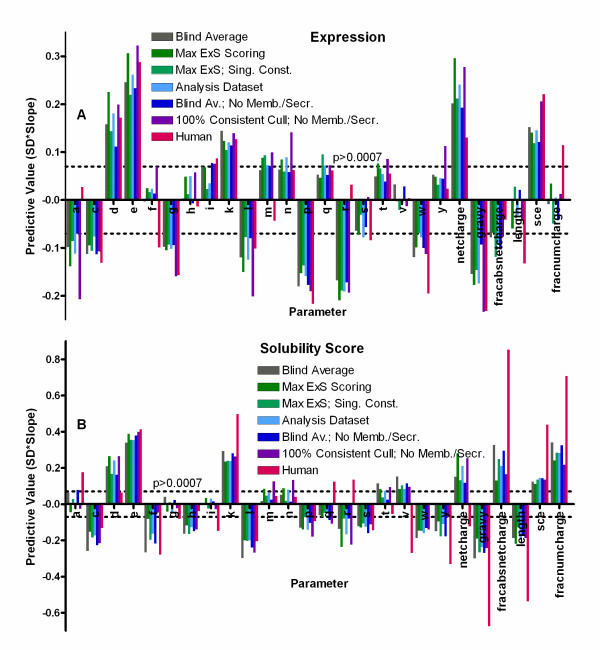
**Statistical analyses of alternative datasets**. Single ordinal logistic regressions equivalent to those performed on the Analysis Dataset in Figure 3 were performed on six alternative datasets. Predictive values (defined in Figure 3) are shown for expression level (panel **A**) and solubility score (panel **B**). The dotted lines indicate the Bonferroni-corrected threshold for significance in the Analysis Dataset (because significance depends on dataset size). The "Blind Average Dataset" contains all NESG protein constructs from the second phase of the Protein Structure Initiative, including multiple constructs for many targets, with scores averaged from all replicate expression trials (19,746 constructs for 13,342 targets). The "Max ExS Dataset" contains exclusively eubacterial proteins from the Analysis Dataset, with the E and S scores taken from the expression trial with the highest E*S value (7,113 constructs for 5,218 targets). The "Max ExS; 1 Construct Dataset" include exactly one construct per target in the Max ExS Dataset (5,218 constructs and targets); the construct with the highest value of E*S was retained for targets with multiple constructs. The "Blind Av.; No Memb./Secr. Dataset" contains proteins from the Blind Average Dataset excluding those predicted by a wider range of metrics to have a transmembrane α-helix or an N-terminal signal peptide or lipopeptide directing secretion out of the cytoplasm (16,888 constructs for 11,698 targets). The "100% Consistent; No Memb./Secr. Dataset" includes only eubacterial proteins with identical E and S scores in all small-scale expression trials and not predicted by any algorithm to have a transmembrane α-helix, N-terminal signal peptide, or lipopeptide (3,633 constructs for 2,583 targets). The "Human Dataset" includes all human proteins from the Blind Average Dataset (3,350 constructs for 1,534 targets). Results from the Analysis Dataset (Figure 3) are shown for comparison.

Six alternative datasets were analyzed using equivalent statistical methods in order to explore the validity and the generality of our results (Figure 4). We compared the results for our principal Analysis Dataset to those for a much larger dataset including expression results from 19,746 constructs for 13,342 targets. This dataset contains a wider range of organisms, including a higher proportion of human proteins (3,350 constructs for 1,534 targets), in addition to having multiple constructs for many targets. Furthermore, rather than taking the maximum E and S scores as was done for the Analysis Dataset, statistical analyses of the larger dataset employed scores set to the average of the E and S values observed in all individual expression trials, leading to use of the label "Blind Average" for this dataset in Figure 4. Analyses were also conducted on a culled version of the Blind Average dataset (labeled "Blind Av.; No Memb./Secr.") that excluded all constructs from proteins that were predicted by a wider variety of methods to have either a transmembrane α-helix or an N-terminal signal peptide targeting the protein for secretion out of the cytosoplasm. A culled version of the Analysis Dataset including only eubacterial targets was also analyzed with the E and S values set to those from the expression trial with the maximum E*S value (labeled "Max ExS"). This last dataset was further culled to remove multiple constructs by selecting the single construct with the highest E*S score, yielding the dataset labeled "Max ExS; 1 Construct," which shows that removing multiple constructs of the same target has little effect on the significant results. An additional highly conservative dataset was analyzed in which the Max ExS Dataset was culled to contain only predicted cytosolic soluble proteins from eubacterial organisms and only constructs giving identical values of E and S in all experimental replicates (labeled "100% Consistent Cull; No Memb./Secr."). One final dataset was analyzed containing only constructs from human proteins in the Max ExS dataset (labeled "Human"). In general, trends of the same sign and similar magnitude were observed in all of these alternative datasets.

**Figure 5:**
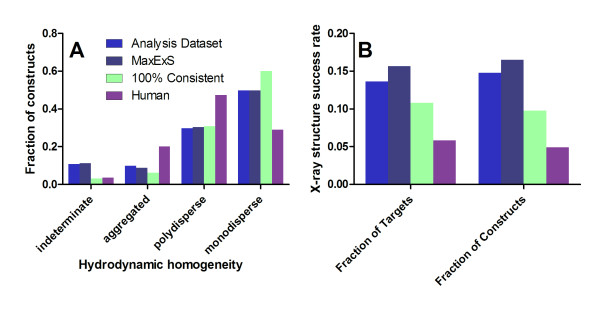
**Biophysical properties of proteins in the different datasets**. The distributions of the hydrodynamic statuses of the concentrated protein stock solutions (panel **A**) and the crystal structure solution rates (panel **B**) are shown for the Analysis Dataset, the Max ExS Dataset, the 100% Consistent Dataset, and the Human Dataset. Hydrodynamic status was determined as previously described [35]; in brief, a thawed aliquot of the final stock solution from each construct was subject to analytical gel-filtration chromatography monitored by in-line static light-scattering and refractive-index detectors. Non-aggregated samples with less than 85% of the protein in a single hydrodynamic species were classified as polydisperse. Crystal structure solution rate is scored based on PDB deposition via the NESG structure-determination pipeline and does not count constructs yielding diffracting crystals unless they were of sufficient quality to support deposition of the structure. Structure solution rates are shown both as fraction of all targets and as fraction of all constructs.

The only notable exception is the Human Dataset. The Human Dataset displays a limited number of significant differences relative to correlations with expression level and a larger number of significant differences relative to correlations with solubility score (Figure 4B*vs*. Figure 3). (Specific discrepancies are described below when relevant to noteworthy trends.) Many different factors could contribute to the broad differences observed in our high-throughput *E. coli *expression trials on predicted human proteins compared to those on predicted bacterial proteins. First, there are greater ambiguities concerning proper identification of open-reading frames in higher eukaryotes [55], due to the presence of introns and other factors, so a larger proportion of the constructs could have improperly defined domain boundaries. Second, this dataset has a much higher proportion of alternative constructs for the same target protein or domain (an average of 2.4 constructs per target *vs*. 1.4-1.5 in the other alternative datasets and 1.0 per target in the Analysis Dataset). Again, this reflects a higher probability of constructs having improperly defined domain boundaries. Furthermore, the protein translation apparatus [56-58] and the chaperone environment [59,60] both have substantial differences in eukaryotic *vs*. bacterial cells, which could also contribute to problems with the folding of the human proteins targets *in vivo *in *E. coli*. Several quality-control measures suggest that such problems are indeed more widespread for the human proteins compared to the bacterial proteins evaluated by NESG. Figure 5A shows that human protein constructs yield monodisperse protein stocks about half as frequently as eubacterial protein constructs (29% in the Human Dataset *vs*. 60% in the 100% Consistent Dataset), while they yield aggregated stocks approximately three times more frequently (20% in the Human Dataset *vs*. 6% in the 100% Consistent Dataset). The rate at which human proteins yielded crystal structures in the NESG pipeline was only one-half to one-third of that in the other datasets analyzed in this paper (Figure 5B). The substantially different solubility-score trends in the Human Dataset compared to the others could be influenced by a greater prevalence of proteins that have not achieved a properly folded native conformation (*i.e.*, that are in a partially or fully unfolded or misfolded state).

### Electrostatic charge has a dominant effect on expression and solubility

Among the evaluated sequence parameters, the most salient effects observed in the Analysis Dataset are from parameters related to electrostatic charge (Figure 3). Considering individual amino acids, the fractional content of three of the charged amino acids, Glu, Asp, and Lys, strongly correlates with higher expression level and solubility score. Notably, these three residues show an influence of similar magnitude on solubility score, while the influence of Lys on expression level is substantially weaker than that of Asp and Glu. (Possible explanations for the systematically different influence of negatively *vs*. positively charged residues on expression level are addressed in the Discussion section below.) Curiously, the fractional content of Arg shows opposite effects to that of Lys, *i.e.*, significant negative correlations with both expression level and solubility score. While Arg does show a positive correlation with solubility score for the human protein dataset, it still shows only approximately a quarter of the magnitude of the positive correlation shown by Lys (Figure 4B), despite the similar electrostatic charge and sidechain entropy of these residues. Despite the contrary effects of arginine, the length-normalized total charge (fraction of Asp + Glu + Arg + Lys, *fracnumcharge*) is the strongest positive predictor of solubility score among the sequence parameters evaluated, while the length-normalized absolute value of net charge (*fracabsnetcharge*) is the second strongest positive predictor among aggregate sequence parameters (right side of Figure 3).

The negative effect of Arg on solubility score in the Analysis Dataset (Figure 3) was surprising and merited further exploration. Arg is encoded in part by rare codons, which are known to impede expression in some cases [16]. Given the strong positive correlation between E and S in our dataset, we hypothesized that such rare codon effects might indirectly cause the negative correlation between Arg content and solubility score. When the fractional content of Arg is split into residues encoded by rare codons *vs*. common codons, Arg residues encoded by the common codons have no significant effect on solubility score. This result contrasts strongly with that observed for Lys, which has a significant positive effect (Additional file 1, Figure S5B). Therefore, we conclude that Arg itself is likely to have some physicochemical or evolutionarily correlated property that reduces solubility score for bacterial proteins, despite its positive charge. Arg residues encoded by both rare and common codons have negative effects on expression (Additional file 1, Figure S5A), although the effect of Arg encoded by rare codons is much more deleterious despite expression in a strain overexpressing the cognate rare tRNAs. These results suggest that Arg exerts a deleterious influence on the expression level of bacterial proteins via both amino acid properties and codon effects.

### Rare codon effects

The significantly different influence of Arg residues encoded by rare *vs*. common codons led us to similarly dissect the influence of rare *vs*. common codons for Ile, Leu, and Pro, the other amino acids known to be encoded in part by expression-reducing rare codons. We observe negative effects on both expression level and solubility score for Leu and Pro. However, surprisingly, Ile showed no significant effect on expression level and a small but significant positive effect on solubility score. To determine whether codon usage influences these results, paired logistic regressions were performed in which residues encoded by rare and common codons were examined independently but simultaneously for each amino acid. Additional file 1, Figure S5 shows these results alongside the comparable single logistic regression results for the total amino acid fractions. In these analyses, the effects observed for residues encoded by common codons should reflect most directly the physicochemical and correlated evolutionary effects of the amino acid itself, while the effects observed for residues encoded by rare codons may also include contributions from inefficient decoding by the cognate tRNAs. Rare codons for Arg, Ile, and Pro all significantly reduce expression (Additional file 1, Figure S5A), and those for Arg and Pro similarly reduce solubility score (Additional file 1, Figure S5B). Ile residues encoded by common codons have a strongly beneficial effect on expression, perhaps via their beneficial influence on solubility score, but the opposing effects of Ile residues encoded by rare *vs*. common codons results in an overall neutral effect of this residue on expression level. The Arg and Ile residues encoded by rare codons seem to impede expression despite overexpression of the cognate tRNAs. These observations indicate that the translation-impeding effects of the rare codons for Arg and Ile likely represent intrinsic properties of the interaction of these codons with the translation apparatus rather than merely a consequence of low expression levels of the cognate tRNAs.

### Hydrophobicity is not a dominant determinant of expression or solubility score

**Figure 6:**
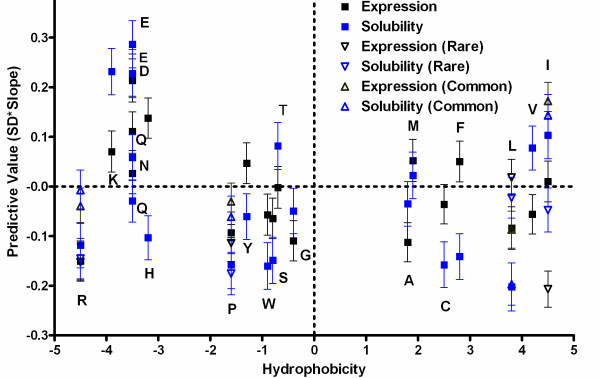
**Predictive values for E and S plotted against amino acid hydrophobicity**. The abscissa gives the hydrophobicity of each amino acid on the Kyte-Doolittle scale. The ordinate gives the predictive values for the fractional content of each amino acid as calculated from single logistic regressions performed against E or S values in the Analysis Dataset. (See the legend to Figure 4 for a definition of the predictive value.) The error bars show 95% confidence. Predictive values for total amino fractions are shown as solid squares. For the four amino acids commonly considered to be encoded in part by rare codons, the predictive values are also shown for the corresponding amino acid fractions encoded by either common codons (triangles pointing up) or rare codons (triangles pointing down); see Additional file 1, Figure S4 and the associated text in the Results section for an explanation of this segregation procedure. Amino acid hydrophobicity is not significantly correlated with amino acid predictive value for expression (p = 0.098) or solubility (p = 0.23).

Several of the results reported above were unexpected. First, Arg, the most hydrophilic amino acid on the Kyte-Doolittle scale, was significantly negatively correlated with solubility score. Second, Ile, the most hydrophobic amino acid on this scale, had a significant positive correlation with solubility score. These observations indicate that the influence of side-chain hydrophobicity on solubility is not straightforward. Figure 6 plots the Kyte-Doolitle hydrophobicity of each amino acid against its predictive value for E and S in our Analysis Dataset. Although mean hydrophobicity exhibits a negative correlation with both expression level and solubility score (Figure 3), this effect comes primarily from the positive influence of the charged residues Asp, Glu, and Lys (Figure 6). The predictive values of the other amino acids are not correlated with hydrophobicity. Of the seven residues with positive hydrophobicity, four have negative effects on solubility, while three have positive effects, including Val and Ile (which are the most hydrophobic residues on some scales). For other amino acids, the effects are uncorrelated with their hydrophobicity. Ala and Gly both have negative effects on expression, which might result from enhanced proteolysis of Ala/Gly-rich sequences, but much weaker and generally neutral effects on solubility score. (While Gly shows a borderline significant effect on solubility score in the Analysis Dataset used to generate Figure 6 -- Gly does not show a consistent effect on solubility score in the broader collection of datasets analyzed in Figure 4, while the clearly significant effects in Figure 6 are all generally consistent in those alternative datasets.) Ser and His both have significant negative impacts on solubility score, but not on expression. Notably, some amino acids, such as Ile and Leu, have different effects despite similar physicochemical properties, including hydrophobicity (as shown on the abscissa in Figure 6). These observations indicate that the stereochemistry of the amino acids makes a strong contribution to their influence on protein solubility score in a large-scale expression dataset.

Additional file 1, Figure S6 presents an equivalent analysis of the relationship between amino acid hydrophobicity and its influence on expression level and solubility score in our Human Dataset. This analysis shows a stronger and more consistent negative correlation between hydrophobicity and solubility score. Notably, the physicochemically similar amino acid pairs that have divergent influences on the Analysis Dataset have consistent influences on the Human Dataset. Arg and Lys both have a positive correlation with solubility score, although the correlation with Arg is substantially weaker than that of Lys (Figure 4B). Similarly, Leu and Ile both show negative correlations, although the correlation with Ile is weaker than that with Leu. Therefore, the influence of amino acid stereochemistry appears to be reduced in this dataset, which shows trends substantially more consistent with the bulk physicochemical properties of the amino acids. However, a substantially smaller proportion of proteins in this dataset are likely to be in their properly folded native conformations, as discussed above (Figure 5), so the observed trends may be influenced by non-native and unfolded protein conformations, where effects are more likely to reflect the bulk physicochemical properties rather than the specific stereochemical features of the amino acids. Further investigations will be required to establish the explanation for the divergent trends observed for human *vs*. bacterial proteins in our high-throughput *E. coli *expression experiments (Figures 4 &6, and Additional file 1, Figure S6).

### Solvent-exposure predictions usefully segregate amino acid parameters

**Figure 7:**
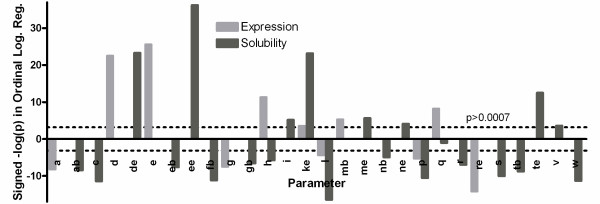
**Segregation of amino acid parameters by predicted surface-exposure vs. burial**. The fractional content of each amino acid was divided according to the localization predicted by the program PHD-PROF. Ordinal logistic regressions were calculated between all sequence parameters listed in Table 1 and E or S values in the Analysis Dataset as described above. Potentially redundant variables (*e.g.*, **a **[total ala] = **ae **[exposed ala] + **ab **[buried ala]) were culled separately for expression and solubility as described in the Methods section. Signed -log(p) values are shown for the parameters that showed a significant correlation with either expression or solubility after parameter culling, using a Bonferroni-corrected p-value threshold of 0.0007.

We hypothesized that the predicted surface-exposure of individual amino acid should significantly influence their effect on solubility score, while this parameter should only have an indirect and therefore weaker influence on expression level. Therefore, we divided the fractional amino acid content by whether the amino acid was predicted by the program PhD/PROF [61] to be buried or surface-exposed, and we ran the same set of ordinal and binary logistic regressions on the divided fractional content of each amino acid. The results of these 72 logistic regressions are shown in Tables S1 & S2. Because some parameters are mathematically related and therefore provide redundant signal (*e.g.*, a = ab + ae), parameter divisions were retained only if buried *vs*. surface-exposed content have statistically significant effects with opposite signs (Figure 7 & Additional file 1, Figure S8). This division of amino acid content shows significant differences for eight amino acids in predicting solubility score, but for only two amino acids in predicting expression level. The positive influence on solubility score of Asp, Glu, and Lys, and to a lesser extent Asn, Met, and Thr, are all derived from surface-exposed residues. Beyond supporting the hypothesis that surface localization critically mediates amino acid influences on solubility, this analysis shows that our analytical approach can provide further insight into differential effects on protein expression *vs*. solubility score, even though the two outcomes are significantly correlated in the dataset.

### Combining parameters to predict experimental outcome

In addition to gaining insight into the primary sequence parameters influencing protein expression and solubility *in vivo*, we sought to develop sequence-based predictors of experimental outcome that could be used to enhance the efficiency of protein-production efforts, especially in high-throughout pipelines. Therefore, based on the statistical analyses reported above, we constructed sequence-based predictors of protein expression level, solubility score, and usability (*i.e.*, of producing a usable protein preparation with E*S > 11). Our software provides substantially more detailed predictions than previous models that report binary outcome probability (*e.g.*, low *vs*. high expression, or the presence *vs*. absence of inclusion bodies). Our software is available online at http://nmr.cabm.rutgers.edu:8080/PES/.

**Figure 8:**
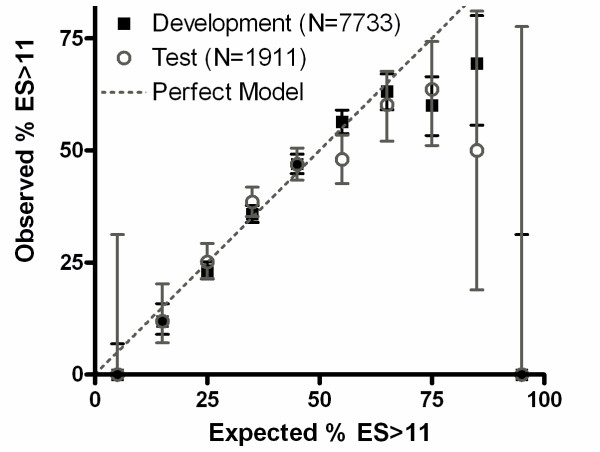
**Performance of a multiparameter model predicting protein usability**. The model employs the significant sequence parameters retained after stepwise multiple binary logistic regression that also fulfill the Akaike Information Criterion (see Methods section). The probability of yielding a protein with E*S > 11 is given by an equation of the form p = 1/(1+exp(-θ)), with θ being a linear combination of the significant sequence parameters. This equation models the results in the Analysis Dataset set closely up to a 65% probability of protein usability (p = 3.7 × 10^-111^, N = 7733) and performs similarly well on a Test Dataset comprising 1911 proteins chosen at random to be excluded from the Analysis Dataset (p = 6.8 × 10^-16^, N = 1911, θ' = 0.85*θ - 0.06). The graph shows the performance of the model based on ten bins at equal intervals of 0.1 in the variable θ. The squares show the fraction of usable proteins in each bin, and the error bars represent 95% confidence limits calculated based on counting statistics.

We used stepwise multiple regressions to create multiparameter models, starting with all significant parameters and removing or re-introducing parameters individually as they became statistically insignificant or regained significance. Because some amino acids showed significantly different effects in single regressions depending on their predicted localization, as documented in the previous section, the total fractional content of these amino acids was divided into the predicted buried and surface-exposed fractions. For example, multiple ordinal logistic regressions were performed using the fractions of the sequence composed of alanine residues predicted to be buried (ab) and exposed (ab) as separate parameters instead of the fraction composed of all alanine residues combined (a). Additional file 1, Table S3 summarizes the slope and significance of the parameters remaining after completion of the culling process on all relevant variables; for comparison to the original set of significantly predictive parameters, the parameters remaining in the usability model are also shown in Figure 8.

In the combined usability metric, called pES for the **p**robability of **E**xpressed and **S**oluble protein, positive effects remain for exposed Gln, exposed Thr, absolute net charge, and, by far the most significant, the fraction of charged residues. Negative effects remain for Cys, buried Phe, Trp, GRAVY, disorder, and, most significant, Arg. Exposed SCE shifts from a positive effect in single regression to a negative effect in the final multiple regression. We hypothesize that SCE initially functions as a proxy for Lys and Glu content; both have high SCE and also carry net electrostatic charge, which improves both solubility and usability. When the effect of electrostatic charge is included explicitly in the multiple logistic regression model via the *fracnumcharge *parameter, the influence of SCE on usability becomes negative, for reasons for that require further study but that are likely to be related to parameter interdependence.

The pES metric models the results observed in the Analysis Dataset closely up to a 65% probability of producing usable protein (p = 3.7 × 10^-111^, N = 7733), and it predicts the results in the corresponding Test Dataset nearly as well (p = 6.8 × 10^-16^, N = 1911) (Figure 8). Using a cutoff of pES > 0.3, the yield of usable proteins could be increased by 13% while keeping 80% of targets; a cutoff of 0.4 would increase yield by 29% while keeping 46% of targets, and a cutoff of 0.5 would increase yield by 45% while keeping 20% of targets. We also developed a usability metric that includes the rare codon effects shown in Additional file 1, Figure S5. This model (Additional file 1, Figure S9) describes the Analysis Dataset somewhat better (p = 9.2 × 10^-137^) than the model based on amino acid sequence without consideration of codon frequency information, and it shows a similar improvement in predicting results in the corresponding Test Dataset (p = 3.3 × 10^-19^).

We also developed separate predictive metrics for expression level and solubility score using the same process of stepwise multiple logistic regression, although these models were constructed using ordinal instead of binary logistic regressions. The slopes and parameters retained in these regressions are reported in Additional file 1, Table S3. They perform very well in predicting the distribution of outcome scores (0-5) observed in both the Analysis Dataset and corresponding Test Dataset (Additional file 1, Figure S10). (Note that in all of our expression and solubility datasets, the mean of the score distributions tends to be near 3, even though this specific value is seldom observed; therefore, ordinal prediction metrics must be evaluated based on the distribution of scores for an ensemble of proteins, as done in Additional file 1, Figure S10, and not merely on the probability-weighted expectation value of the score for a single protein.)

### Permissive vs. enhancing sequence parameters

To deepen understanding of the related mechanistic effects, we examined the impact of individual parameters more closely to determine whether some parameters influenced outcomes at the low end of the score range (*i.e.*, no expression (E = 0) *vs*. any expression at all (E>0) -- "permissive" factors) or at the high end of the range (*i.e.*, very high expression (E = 5) *vs*. lesser expression (E<5) -- "enhancing" factors). Many parameters have such disparate impacts (Additional file 1, Figure S11). Notably for expression, parameters related to the factional content of charged or hydrophobic residues are primarily permissive, while net charge is primarily enhancing. Similar patterns exist for solubility, but in this case most significantly permissive factors were also significantly enhancing. For more details, see the SI.

### Opposing parameter influence on expression level and solubility score vs. crystallizability

**Figure 9:**
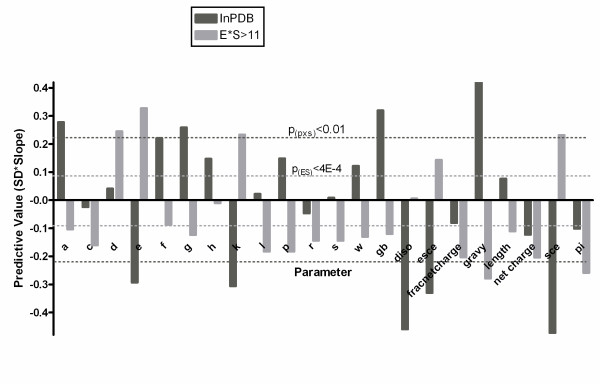
**Opposing influence of sequence parameters on protein expression/solubility vs. crystallization propensity**. Predictive values for usability (*i.e.*, having E*S > 11) were calculated using binary logistic regression against the Analysis Dataset, while predictive values for the propensity to yield a crystal structure were calculated using binary logistic regression against crystallization results obtained from a subset of the same proteins in the NESG pipeline, as previously reported [35]. Predictive values (defined in the legend for Figure 4) are compared because they normalize for the large difference in the sample sizes in the expression and crystallization datasets (9,866 *vs*. 679 proteins), which prevents comparison based on measures of statistical significance. Parameters are shown if significant at the indicated Bonferroni-corrected p-value thresholds in either analysis.

Previous studies noted that factors predicting crystallizability of expressed, soluble proteins were less effective in predicting success in going from gene sequence to crystallized protein [35]. This observation suggested that some sequence parameters have opposing effects on expression or solubility *vs*. crystallizability. The current analyses unfortunately confirm this inference. Almost every parameter that has significant positive influence on usability has a negative influence on crystallizability according to our previous published crystallization datamining studies [35], and vice versa (Figure 9). To some extent, these observations are mechanistically tautological because crystallization represents orderly precipitation of a protein out of an aqueous solution. Nonetheless, they highlight the difficulty inherent in attempting to use single amino-acid substitutions to engineer improved protein crystallization behavior, as noted in prior reports of solubility problems encountered in the course of such mutagenesis experiments [62].

These opposing effects of primary sequence parameters on expression/solubility *vs*. crystallizability are reflected in the distribution of pES scores among structures deposited into the PDB by the NESG. Proteins which yielded X-ray crystal structures had significantly higher usability scores than the full set of targets evaluated, but significantly lower scores than proteins yielding NMR solution structures (Additional file 1, Figure S12). The most likely explanation for these trends is that proteins suitable for X-ray structure determination must meet at least minimal expression and solubility requirements, and therefore tend to have somewhat higher pES, but proteins that exhibit the exceptionally high solubility required for NMR studies and have correspondingly higher average pES are enriched in high-entropy Glu and Lys residues, making them less likely to crystallize successfully. In evaluating these trends, it is important to note that proteins with E*S < 12 are more difficult to produce in high yield and were therefore dropped from the NESG protein-production pipeline. The observed bias towards higher usability scores may not be as strong outside of a high-throughput pipeline. Overall, selection of targets with medium-to-high predicted usability scores would be expected to increase the likelihood of producing proteins suitable for X-ray crystal-structure determination, while selection of targets with very high usability scores would be expected to increase the likelihood of producing proteins suitable for NMR solution-structure determination.

### Predicting NMR success

This last conclusion is reinforced by explicit efforts to analyze the primary sequence correlates of successful NMR structure determination, which are detailed in the SI. These studies led to the conclusion that the characteristics necessary for obtaining expressed, soluble protein are the most important determinants of the probability of obtaining an NMR structure solution (Additional file 1, Figure S13). Therefore, the usability predictor described above can also function as a predictor of suitability for NMR structure determination.

## Discussion

### General considerations in interpreting observed correlations

Care must be exercised in interpreting correlations between sequence parameters and experimental observations in a large-scale biological dataset. While such correlations could derive from a direct mechanistic effect of the correlated sequence parameter, they could alternatively derive from simultaneous evolutionary selection of two different physical properties that do not have a direct mechanistic relationship. Examples of such alternative hypotheses are presented when discussing specific correlations below. In all such cases, additional analyses, ideally both informatic and experimental, will be required to determine the relative contributions of direct *vs*. indirect mechanisms to the observed large-scale correlations.

### Strong correlation between expression level and solubility score

One salient feature of our large-scale expression dataset is a strong positive correlation between expression level and solubility score (Figure 1 & Additional file 1, Figure S14) for protein-coding genes from diverse phylogenetic sources. A similarly strong positive correlation between equivalent parameters was observed for endogenous *E. coli *genes in a large-scale study of cell-free *in vitro *translation [34], and a reanalysis of these data indicates this correlation remains strong if the solubility results from *in vitro *translation are compared to endogenous protein expression levels *in vivo *in *E. coli *as determined using quantitative mass spectrometry methods (G. Boel, L. Brown, and J.F. Hunt, unpublished results). Furthermore, proteins whose mRNAs are expressed at a high level in *E. coli *have been demonstrated to have a reduced tendency to form amyloid aggregates [12]. Therefore, the strong correlation between E and S observed in our large-scale dataset appears to be a conserved feature of the expression of naturally evolved genes, at least in *E. coli*.

One possible explanation for the strong positive correlation between E and S would be an indirect evolutionary correlation between these parameters. Highly expressed proteins, which are known to have more strongly conserved amino acid sequences [63-65], may be constrained to have more robust folding and solubility properties because of their higher cellular concentration. In this case, the parameters influencing expression level could be completely unrelated to the amino acid sequence of the protein (*e.g.*, mRNA structure and codon usage), but its amino acid sequence could be independently but simultaneously selected to promote solubility.

Alternatively, there could be some kind of direct mechanistic coupling between the processes influencing protein expression and solubility. For example, aggregation of nascent proteins could inhibit their translation. Additionally, aggregated proteins could be targeted preferentially for degradation, thereby reducing their steady-state expression level. While such explanations are plausible, many mutations that impair solubility do not reduce expression [52], indicating that solubility does not directly influence expression level in all cases. Another possibility for mechanistic coupling is that more rapid protein translation, which would tend to produce higher steady-state expression levels, could promote more efficient folding and thereby promote higher solubility for some proteins. This possibility is supported by the observation of an enhanced content of more efficiently translated codons at buried and aggregation-prone sites in proteins [66,67]. On the other hand, removal of translational pause sites from some proteins leads to impaired folding and decreased solubility, presumably due to a decrease in folding efficiency when translation is accelerated [68]. Furthermore, an improved yield of soluble protein is frequently obtained by lowering the growth temperature of *E. coli*, which slows protein translation, and mutations that reduce ribosomal translation speed in *E. coli *have been shown to improve the soluble yield of some proteins [69]. However, such perturbations broadly modulate the physiological state of the cell, and a temperature change also modulates the physicochemical properties of proteins and even water. Further investigation will be required to understand the mechanisms underlying these seemingly divergent effects and whether more rapid translation generally promotes or impedes more efficient protein folding *in vivo*.

Therefore, many explanations are possible for the strong positive correlation between protein expression level and solubility score observed in our large-scale dataset. Given the evidence that this correlation is a general property of naturally evolved protein-coding genes [12,34], at least when expressed in *E. coli*, elucidating the source of this correlation should be an important goal of future studies.

### Influence of electrostatic charge on expression level

The fractional content of charged (acidic and basic) amino acids is a dominant predictor of good protein solubility in all of the large-scale protein expression datasets examined in this study (Figures 3 and 4B). Consistent with the generally strong positive correlation between expression level and solubility score observed in these datasets (Figure 1 & Additional file 1, Figure S14), Asp and Glu have a similarly strong positive correlation with expression level (Figures 3 &4A). However, Lys shows a much weaker positive correlation with expression than Asp or Glu, despite its equivalently strong positive correlation with solubility score, and Arg shows a negative influence on expression, even when coded by common codons (Additional file 1, Figure S5). Given the strong correlation between expression level and solubility score, and the statistical and likely mechanistic dominance of charge on solubility, the simplest explanation for these observations is that positively charged residues reduce translation efficiency while negatively charged residues do not. Such an effect could derive from electrostatic attraction of the positively charged amino acids to rRNA [70]. Indeed, one specific Arg codon has been demonstrated to slow translation [71]. However, alternative explanations cannot be ruled out, including a differential influence of positive *vs*. negative charges on protein degradation rates. Further mechanistic study will be required to distinguish between possible explanations.

### Amino acid influence on protein solubility

Surprisingly, some amino acids with very similar physicochemical properties (Lys *vs*. Arg and Leu *vs*. Ile/Val) have divergent influences on solubility score in all of our protein expression datasets except the Human Dataset (Figures 3,4), which is likely to have a substantially higher fraction of disordered or improperly folded proteins (Figure 5). Furthermore, excluding the influence of Asp, Glu, and Lys, there is no correlation between hydrophobicity and influence on solubility score in our primary Analysis Dataset, although there is in the Human Dataset. The positive influence of some residues on solubility score could potentially represent a substitution effect (*e.g.*, Ile or Val being less deleterious than Leu at positions constrained to be hydrophobic). However, even an effect of this kind is not readily rationalized based on the bulk thermodynamic properties of the individual amino acids. These observations suggest that, for the large and phylogenetically diverse set of bacterial proteins included in our large-scale expression experiments, the stereochemical properties of the amino acids significantly influence protein solubility during *in vivo *expression in *E. coli*.

Solubility following expression *in vivo *could be strongly influenced by the thermodynamic stability of the protein construct, because unstable proteins often have enhanced misfolding and aggregation propensity. While stability is expected to depend primarily on local stereochemical interactions in the folded state of the protein, previous work documenting sequence bias in proteins from hyperthermophilic organisms has suggested that the content of a specific set of amino acids (IVYWREL -- Ile, Val, Tyr, Trp, Arg, Glu, and Leu) may be generally correlated with increased protein stability [27]. However, the trends observed in the sequences of hyperthermophilic proteins do not match those observed in our analyses of solubility score. Fractional content of Ile, Val, and Glu are correlated with higher solubility score in our datasets (other than the Human Dataset), while fractional content of Tyr, Trp, Arg, and Leu are correlated with a lower solubility score. Therefore, either the amino acids IVYWREL are not correlated with higher protein stability at mesophilic temperature or stability effects do not account for the trends in solubility score observed in our datasets.

An alternative hypothesis is that the observed solubility-score correlations could reflect preferential participation of some amino acids in stable interprotein interaction interfaces. In the absence of the physiological binding partner, the exposure of such interfaces to solution might tend to drive aggregation. While such an effect would still reflect a greater propensity for amino acids with specific stereochemical properties to mediate interprotein contacts, it is nonetheless important to understand whether this propensity depends on being present in an evolved interprotein interaction surface. In this case, the statistical influence of the amino acid could represent in part a surrogate for the presence of unfulfilled interprotein interaction interfaces. Therefore, we examined the relationship between the predictive values for outcome in our Analysis Dataset and two parameters related to the participation of each amino acid in stable interprotein interaction interfaces in the Protein Protein Data Bank (PDB), as identified by the BIOMT [72] flag in crystal structures deposited in the PDB (V. Naumov and J.F. Hunt, manuscript in preparation). The first of these parameters is the frequency of the amino acid relative to all amino acids participating in such interfaces (Additional file 1, Figure S7A), while the second is the overrepresentation ratio for each amino acid in such interfaces compared to an appropriately surface-weighted distribution of the protein sequences yielding the structures (Additional file 1, Figure S7B). Neither of these parameters shows a significant correlation with the complete set of predictive values observed in our Analysis Dataset. Therefore, participation in stable interprotein interaction interfaces is not a dominant determinant of amino acid influence on expression level or solubility score in this dataset. However, some noteworthy points concerning the influence of specific amino acids emerge from this analysis.

Notably, Leu and Arg, which both show a disproportionately deleterious influence on solubility compared to amino acids with similar bulk physicochemical properties, are the most frequent amino acids making contacts in stable interprotein interaction interfaces (Additional file 1, Figure S7A). This observation suggests that these amino acids have an enhanced interaction potential due to their stereochemical properties. In this context, converting these amino acids to less interaction-prone residues with similar bulk physicochemical properties might help reduce non-specific interprotein interaction and aggregation (as discussed in more detail below.)

Arg is also the most strongly overrepresented amino acid in stable interprotein interaction interfaces compared a properly surface-weighted distribution of the amino acid sequences yielding crystal structures in the PDB (Additional file 1, Figure S7B). In comparison, the other charged amino acids (Lys, Glu, and Asp), are all slightly underrepresented in such interfaces. Therefore, the surprisingly deleterious influence of Arg on solubility score and its divergent influence compared to Lys, Glu, and Asp could reflect in part a surrogate effect related to formation of stable interprotein complexes, because sequences forming such interfaces should have an enhanced content of Arg. However, the mechanistic underpinning of this surrogate effect would be the greater interaction propensity of Arg compared to the other charged amino acids, perhaps due to the high H-bonding potential of its guanidino group.

In contrast, the discrepancy between the influence of Leu *vs*. Ile or Val on solubility score seems unlikely to be attributable to such a surrogate effect and likely to be directly attributable to its stereochemical properties. While Leu is the most frequent amino acid in BIOMT interfaces (Additional file 1, Figure S7A), it is also the most abundant residue overall, and it is slightly depleted in BIOMT interfaces compared to its frequency in an appropriately surface-weighted amino acid distribution (Additional file 1, Figure S7B). While Ile and Val are slightly more depleted, the observation that Leu is depleted from such interfaces makes its enrichment in a protein sequence unlikely to be a surrogate for formation of stable interprotein interaction complexes. It is possible that the stereochemical features of Leu, perhaps its knob-like hydrophobic structure, give it a stronger tendency to mediate non-specific interprotein interactions compared to the residues with similar bulk physicochemical properties, which could contribute to its comparatively deleterious influence on protein solubility in our large-scale expression datasets. The observation that Leu is the most abundant residue in stable interprotein interaction interfaces (Additional file 1, Figure S7A) establishes its strong tendency to mediate specific interprotein interactions.

### Potential implications for protein expression and engineering

The computational predictors we have developed for expression and solubility score can increase the likelihood of expressing high quantities of soluble proteins. Target selection necessitates a tradeoff between a higher rate of success with retained targets and discarding a higher proportion of the initial set. Using our metric with a reasonable cutoff of pES>0.4, we would expect a 29% increase in usable targets while discarding 54% of the pool. This approach could prove useful for high-throughput studies.

Our results also have implications relative to strategies for engineering individual proteins to improve their expression and solubility characteristics. They provide statistical justification for adding Lys, Gln, and Glu residues and deleting disordered segments to improve yield of soluble protein and suitability for NMR solution-structure determination, strategies that have been pioneered in previous studies [6,7]. Our results also suggest new approaches to be evaluated for their efficacy in increasing the expression and solubility of individual proteins.

The more favorable influence of Ile and Val on solubility score compared to the very deleterious influence of Leu suggests that substituting Leu with Ile or Val at some protein positions might produce an improved yield of soluble protein. Given the uncertain mechanistic origin of the statistical trends in our expression datasets, it is unclear whether or how generally such substitutions will have beneficial effects. However, engineering experiments of this kind represent one approach to clarifying the physicochemical effects accounting for the observed large-scale correlations. Similar logic suggests that some Arg-to-Lys substitutions might also improve yield of soluble protein. However, outcome in this case is even more uncertain because the deleterious statistical influence of Arg on solubility score could represent a surrogate effect reflecting the influence of unbound interprotein interaction surfaces, in which case single amino acid substitutions are less likely to solve related solubility problems. Additional informatics analyses and biochemical experimentation will be required to elucidate the mechanisms underlying the correlations observed in our large-scale experimental dataset and whether any of them can be applied productively to engineering improved protein expression results.

## Materials and methods

### Target selection and classification

For the Analysis Dataset, 9644 protein target sequences expressed between 2001 and June 2008 were selected from the SPINE database [73]. Protein sequences were randomly assigned at a 4:1 ratio (7733:1911) to the Analysis Dataset or the corresponding Test Dataset. Proteins with transmembrane α-helices predicted by TMMHMM [74] (*i.e.*, >20% low-complexity sequence) were excluded from the pipeline, and therefore were not included in our analyses. A small fraction of constructs that entered the pipeline have predicted lipoprotein signal sequences (239, ~2.5%) [75] that were not identified at the time of target selection. Another small fraction have predicted signal sequences (622, ~6.4%) [76], but many of these were expressed in soluble form following export to the periplasm and cleavage of the signal peptide (some of which gave crystal structures). Because the expression and solubility-score distributions for these proteins were very similar to that of the entire dataset (Additional file 1, Figure S14), they were retained in the Analysis Dataset. However, to explore the possible influence of such proteins and other factors related to protein source and data processing on overall trends, the basic sequence analyses were repeated on six alternative datasets, as described in the Results section and shown in Figure 4.

### Protein expression & purification

Proteins were expressed, purified, and analyzed as previously described [35,45], including assessment of protein aggregation state using analytical gel-filtration chromatography with static light-scattering and refractive index detectors. Small-scale expression scores were used for all analyses (other than the comparison of these results with those from corresponding large-scale purification experiments as shown in Additional file 1, Figure S1B).

### Datamining variables

Datamining analyses were conducted on native sequences with tags removed. Three outcome variables were considered: independent 0-5 integer scores for expression (E) and solubility (S), as evaluated by Coomassie-stained gel electrophoresis, and the binary variable of usability, defined as having a product of expression and solubility scores greater than 11 (*i.e.*, 12 or higher given that E and S are both constrained to be integers). Input variables included the frequency of each amino acid, either total or predicted by the program PHD/PROF [61] to be buried or exposed (60 variables in total), and the compound sequence metrics of charge, pI, grand average of hydrophobicity (GRAVY), Monte Carlo sidechain entropy (SCE), fraction of residues predicted to be disordered (DISOPRED), and the length of the construct sequence. The number and fraction of charged residues were also evaluated, calculated as signed or unsigned sums of the frequencies of appropriate combinations of Arg, Lys, Glu, and Asp residues considered as both whole and fractional values. Isoelectric point was calculated at ExPASy (http://ca.expasy.org/tools/pi_tool.html). GRAVY was calculated using the Kyte-Doolittle hydropathy parameters [54]. The Creamer scale [77] was used for the SCE values of the individual amino acids. DISOPRED scores were calculated using DISOPRED2 [78] with a 5% false positive rate. Mean exposed SCE was calculated as the mean for all residues predicted to be exposed, while all calculations based on secondary structure class used total chain length as the denominator.

### Statistical methods

Logistic regressions were performed in STATA (Statacorp, College Station, TX) with significance determined from Z-scores for individual variables and chi-squared distributions for multi-parameter models. For each of the three outcome variables (expression, solubility, and usability), single logistic regressions were run to evaluate potential correlations between the outcome variable and the 72 input variables calculated from the protein sequence. Proportional odds ordinal logistic regressions were used for expression and solubility, and binary logistic regression for usability [53]. In binary logistic regression, the probability of a positive outcome is given by the function Pr(Y = 1) = e ^**θ**^/(1+e^**θ**^), where **θ **is the linear combination of predictive variable values and their slopes. For ordinal logistic regression, the probability that the outcome is less than or equal to a value *j *is given by the function P(Y≤*j*) = e^(t*j*-^**θ**^)^/(1+e^(t*j*-**θ**)^, with the added parameter t*j*, a threshold value for each value of the outcome variable. Among the three variables for each amino acid (total fraction, predicted buried fraction, and predicted exposed fraction), the buried/exposed variables were used if they had opposite-signed slopes in single logistic regressions, otherwise the total fraction was used. For charge variables, the more significant of the whole or fractional versions of each variable was kept. All variables which were not significant at the Bonferroni-adjusted p-value of 0.00069 (0.05/72) were dropped. Combined models were built by stepwise forward/reverse logistic regression with p-value cutoffs of 0.05 for removal and 0.049 for addition. Each variable in the resulting model was individually removed to check for improvement in Akaike's Information Criterion (AIC) [79]. Any variable whose removal improved the AIC was discarded from the model. Counting-statistics-based 95% confidence intervals were calculated using Bayesian maximum likelihood estimates of the binomial distribution.

## Competing interests

The authors declare that they have no competing interests.

## Authors' contributions

WNP helped conceive this study, carried out most statistical analyses, performed stability studies, and drafted the manuscript. SKH, SNT, JKE, JDL, and PM assisted with database development and statistical analyses. AB helped design the statistical analyses. VN performed PDB dataming studies. TA, RX, BR, JFH, and GTM oversaw organization and operation of the NESG protein-production pipeline. JFH helped conceive this study, participated in its design, and helped write the manuscript. All authors approved the final manuscript.

## Supplementary Material

Additional file 1 **Supplemental Information**. Additional file 1 includes Supplemental Figures and Tables as referenced in the text, in addition to Supplemental Methods relating to crystallization and NMR solution-structure determination. Supplemental Text describes in greater detail the analysis of permissive *vs*. enhancing factors and the prediction of successful NMR solution-structure determination [83-87]. Click here for file
