# Supplementary material for: Large-scale experimental studies show unexpected amino acid effects on protein expression and solubility in vivo in E. coli
Source: Microb Inform Exp. 2011 Jun 27;1:6. doi: 10.1186/2042-5783-1-6 (PMC3372292; doi:10.1186/2042-5783-1-6)
Supplement: Additional file 1 — Supplemental Information. Additional file 1 includes Supplemental Figures and Tables as referenced in the text, in addition to Supplemental Methods relating to crystallization and NMR solution-structure determination. Supplemental Text describes in greater detail the analysis of permissive vs. enhancing factors and the prediction of successful NMR solution-structure determination [83-87]. [file 2042-5783-1-6-S1.DOC]

***Supplemental Information for***

**Large-scale experimental studies show unexpected amino acid effects on protein expression and solubility *in vivo* in *E. coli***

**W Nicholson Price II^1,2^, Samuel K Handelman^1,2^, John K Everett^1,3^,**

**Saichiu N Tong^1,3^, Ana Bracic^4^, Jon D Luff^1,2^, Victor Naumov^1,2^,**

**Thomas Acton^1,3^, Philip Manor^1,2^, Rong Xiao^1,3^, Burkhard Rost ^1,5^,**

**Gaetano T Montelione^1,3,6^, and John F Hunt^1,2^***

^1^Northeast Structural Genomics Consortium;

^2^Department of Biological Sciences, Columbia University, 702A Fairchild Center, MC2434,

1212 Amsterdam Avenue, New York, 10027, USA;

^3^Department of Molecular Biology and Biochemistry, Center for Advanced Biotechnology and Medicine, Rutgers University, 679 Hoes Lane, Piscataway, 08854, USA;

^4^Wilf Family Department of Politics, New York University,

19 W. 4^th^ Street, New York, 10012, USA;

^5^Department of Biochemistry and Molecular Biophysics, Columbia University,

1130 St. Nicholas Avenue, New York, 10032, USA;

and

^6^Department of Biochemistry, Robert Wood Johnson Medical School, University of Medicine and Dentistry of New Jersey, 679 Hoes Lane, Piscataway, 08854, USA.

***** Corresponding author: (212)-854-5443 voice; (212)-865-8246 FAX; jfhunt@biology.columbia.edu.

Email addresses:

WNP: wnp2101@columbia.edu

SKH: samuel.handelman@gmail.com

JKE: everett.jk@gmail.com

SNT: tong@cabm.rutgers.edu

AB: anabracic@gmail.com

JL: jl3199@columbia.edu

VN: mutantgene@gmail.com

TA: acton@cabm.rutgers.edu

PM: pmanor@biology.columbia.edu

RX: rxiao@cabm.rutgers.edu

BR: rost@columbia.edu

GTM: guy@cabm.rutgers.edu

JFH: jfhunt@biology.columbia.edu

**Supplemental Text**

***Details regarding analyses of permissive vs. enhancing sequence parameters.*** We evaluated whether some sequence parameters have a differential influence across the range of observed expression and solubility score values. A parameter can potentially show an equivalent effect across the entire score range; in this case, an increase in the parameter would have the same influence on the probability of scoring 0 *vs.* 1 for expression as for scoring 3 *vs.* 4 for expression. Alternately, factors could operate differently at different ends of the score spectrum. For instance, some sequence parameters, which we dub “permissive”, could have a large influence on whether a protein scores 0 *vs.* any higher value but less influence on the distribution of scores above 0. Alternatively, other sequence parameters, which we dub “enhancing”, could have a large influence on whether a protein scores 5 *vs.* any lower value but less influence on the distribution of scores below 5. Framing this issue mathematically involves evaluating whether the slopes of the paired binary logistic regressions between adjacent ordinal score values change significantly over the observed score range. Therefore, for every significant sequence parameter, we calculated the Brant statistic [83], which evaluates the likelihood that the slopes between different outcome steps in an ordinal logistic regression are equal, and we also ran simple binary logistic regressions based our definitions of permissive (0 *vs.* 1-5) and enhancing (0-4 *vs.* 5). Fig. S4 shows signed ‑log(p) values for these regressions for all factors which were significant predictors of expression or solubility score, sorted by the significance of their Brant statistic.

The majority of expression-predicting parameters differed significantly in their influence across the range of E values. Permissive sequence parameters include the fractional number of charges, SCE, fractional exposed Lys content, exposed SCE, and fractional Glu content. Parameters opposing permissiveness include GRAVY and fractional Pro, Leu, Gly, and Ala content. Enhancing sequence parameters include the fractional Asp, buried Met, and His content. Parameters opposing enhancement include net charge, fractional predicted backbone disorder, fractional exposed Arg content, and fractional absolute net charge. Gln showed no significant difference in its influence on expression throughout the range of E scores, and a few parameters (GRAVY, net charge, and fractional Glu, exposed Arg, Asp, and Ala content) showed significant effects that differed substantially in magnitude over the score range. No parameter had an opposite influence on permissiveness *vs.* enhancement.

For solubility score, fractional predicted backbone disorder and fractional exposed Gln content are permissive but not enhancing. All other sequence parameters had significant effects on both permissiveness and enhancement. SCE, exposed SCE, fractional exposed Lys content, and fraction of charged residues were primarily permissive, while GRAVY, chain length, and fractional buried Gly, buried Phe, buried Thr, Cys, and Ile content primarily opposed permissiveness. Fractional exposed Asp content was primarily enhancing, while net charge and fractional Arg content primarily opposed enhancement. All other significant predictors had a similar influence on permissiveness *vs.* enhancement.

***Analyses of sequence parameters influencing suitability for NMR structure determination.*** Nearly 1,000 protein constructs less than 200 amino acids in length that were expressed and soluble at a sufficient level were screened for their suitability for NMR solution-structure determination by the NESG. Heteronuclear ^1^H-^15^N single quantum correlation (HSQC) spectra acquired from these proteins under standard condition were subjectively scored as unfolded, poor, promising, good, or excellent [45, 84, 85]. After converting evaluations from “poor” to “excellent” into integer scores from 1-5 (*i.e.*, 1 for unfolded to 5 for excellent), the same ordinal logistic regression methods described in the main text were used to evaluate possible correlations between primary sequence parameters are NMR spectral quality. After single regressions and parameter culling (Fig. S13A), significant positive effects were observed for fractional exposed Thr and buried Trp content. Significant negative effects were observed for protein chain length, the number of charged residues, and fractional buried Thr content. However, when the parameters were combined into a predictive model using the stepwise ordinal logistic regression procedure described in the main text, only protein chain length and fractional exposed Thr and buried Trp content remained significant (Fig. S13A). The significant influence of the number of charged residues observed in single ordinal logistic regression is likely a surrogate for the statistically dominant influence of protein chain length, which is strongly positively correlated with the number of charged residues. The overall multiple logistic regression model accurately describes the development set of 781 proteins (p = 1.5x10^11^) but shows only marginal significance in predicting outcome in the corresponding test set of 201 proteins (p = 0.07) (Figs. S13B & C). We hypothesize that the most important sequence parameters influencing suitability for NMR solution-structure determination are equivalent to those influencing the probability of obtaining a useable protein preparation (*i.e.*, with E*S ≥ 11), so that simple sequence parameters only have a relatively insignificant influence on the probability of obtaining an excellent NMR spectrum once a usable protein sample had been obtained.

**Supplemental Materials and Methods.**

***Crystallization.*** Initial high-throughput crystallization screening was conducted using the 1536 well microbatch robotic screen at the Hauptmann-Woodward Institute [35, 86]. Proteins failing to yield rapidly progressing crystal leads were subjected to vapor diffusion screening, typically 250-300 conditions (Crystal Screens I & II, PEG-Ion, and Index screens from Hampton Research or equivalent screens from Qiagen) at both 4˚ C and 20˚ C. These latter screens were conducted in the presence of substrate or product compounds if commercially available. Crystal optimization, diffraction data collection at cryogenic temperatures, structure solution using single or multiple-wavelength anomalous diffraction techniques, and refinement were conducted using standard methods [35, 85].

***NMR structure solution.*** NMR structure solution was performed as previously described [84].

**Table S1. *Single logistic regressions on fractional amino acid content.*^a^**

|  | **Expression** | | **Solubility score** | | **Usability** | |
| --- | --- | --- | --- | --- | --- | --- |
| **Parameter** | **Slope** | **P-Value** | **Slope** | **P-Value** | **Slope** | **P-value** |
| a | -3.07 | **1.27E-08** | -0.96 | 0.119 | -2.71 | **9E-06** |
| ab | -4.83 | **6.3E-08** | -5.88 | **7.04E-09** | -8.09 | **2.19E-15** |
| ae | -2.44 | 0.0009 | 2.20 | 0.0083 | 0.45 | 0.582 |
| c | -2.54 | 0.069 | -11.1 | **6.89E-12** | -11.2 | **3.17E-10** |
| cb | -2.58 | 0.093 | -9.94 | **1.7E-08** | -10.4 | **1.61E-07** |
| ce | -3.73 | 0.384 | -26.1 | **8.8E-08** | -22.9 | **5.12E-06** |
| d | 10.4 | **6.2E-23** | 11.06 | **8.76E-21** | 12.3 | **4.18E-25** |
| db | 15.3 | **7.82E-05** | -8.78 | 0.039 | -3.33 | 0.441 |
| de | 9.65 | **2.97E-19** | 12.1 | **9.19E-24** | 13.0 | **5.93E-27** |
| e | 8.14 | **5.08E-26** | 10.4 | **3.55E-33** | 12.0 | **1.34E-42** |
| eb | 12.3 | 0.029 | -33.9 | **4.25E-08** | -21.6 | **0.0007** |
| ee | 7.80 | **2.44E-24** | 10.9 | **1.12E-36** | 12.2 | **1.18E-44** |
| f | 2.90 | 0.014 | -8.14 | **9.36E-10** | -4.99 | **0.0002** |
| fb | 3.05 | 0.017 | -9.76 | **1.2E-11** | -6.71 | **3.84E-06** |
| fe | 1.84 | 0.529 | 1.41 | 0.674 | 4.12 | 0.204 |
| g | -4.32 | **5.96E-08** | -1.96 | 0.030 | -4.78 | **1.22E-07** |
| gb | -0.82 | 0.465 | -6.40 | **4.9E-07** | -6.56 | **3.06E-07** |
| ge | -5.97 | **1.28E-09** | 1.93 | 0.084 | -2.33 | 0.037 |
| h | 10.1 | **9.76E-12** | -7.56 | **3.48E-06** | -0.75 | 0.645 |
| hb | 12.5 | **3.16E-06** | -12.3 | **2.92E-05** | -5.50 | 0.067 |
| he | 9.51 | **1.61E-07** | -5.66 | 0.0044 | 1.35 | 0.502 |
| i | 0.39 | 0.624 | 4.06 | **1.24E-05** | 3.14 | **0.0005** |
| ib | 1.49 | 0.101 | 3.44 | 0.001 | 2.90 | 0.0042 |
| ie | -4.95 | 0.015 | 8.54 | **0.0003** | 5.66 | 0.013 |
| k | 1.99 | 0.0006 | 6.56 | **3.77E-23** | 6.67 | **1.69E-23** |
| kb | -2.84 | 0.741 | -9.32 | 0.342 | -12.8 | 0.186 |
| ke | 2.03 | **0.0005** | 6.67 | **1.25E-23** | 6.83 | **3.31E-24** |
| l | -2.93 | **8.49E-05** | -7.07 | **6.83E-17** | -6.56 | **9.19E-15** |
| lb | -2.40 | 0.0025 | -7.22 | **1.35E-15** | -6.53 | **4.83E-13** |
| le | -3.61 | 0.020 | -3.20 | 0.069 | -3.87 | 0.029 |
| m | 4.06 | 0.014 | 1.73 | 0.342 | 0.60 | 0.741 |
| mb | 9.08 | **1.03E-05** | -5.78 | 0.010 | -3.66 | 0.111 |
| me | -4.05 | 0.103 | 12.9 | **4.43E-06** | 6.59 | 0.016 |
| n | 1.25 | 0.201 | 2.79 | 0.012 | 2.77 | 0.011 |
| nb | 2.04 | 0.569 | -17.2 | **2.24E-05** | -17.2 | **2.14E-05** |
| ne | 1.19 | 0.242 | 4.38 | **0.0001** | 4.38 | **0.0001** |
| p | -4.25 | **9.42E-06** | -7.19 | **5.03E-11** | -8.52 | **2.17E-14** |
| pb | -1.96 | 0.395 | -21.7 | **3.46E-17** | -20.1 | **1.72E-14** |
| pe | -4.67 | **8.2E-06** | -3.91 | 0.0011 | -5.84 | **1.44E-06** |
| q | 5.47 | **1.2E-08** | -1.44 | 0.171 | 3.06 | 0.0043 |
| qb | 8.22 | 0.057 | -21.0 | **1.24E-05** | -15.9 | 0.0011 |
| qe | 5.24 | **7.87E-08** | -0.45 | 0.674 | 3.95 | **0.0003** |
| r | -5.13 | **8.65E-14** | -4.04 | **2.1E-07** | -4.93 | **1.2E-09** |
| rb | 2.53 | 0.484 | -11.6 | 0.0039 | -9.57 | 0.018 |
| re | -5.40 | **1.16E-14** | -3.72 | **2.48E-06** | -4.74 | **1E-08** |
| s | -2.90 | 0.0017 | -6.72 | **1.66E-10** | -6.55 | **1.06E-09** |
| sb | -1.22 | 0.522 | -15.6 | **3.87E-13** | -15.4 | **1.44E-12** |
| se | -2.77 | 0.0036 | -3.17 | 0.0033 | -2.99 | 0.0063 |
| t | -0.09 | 0.928 | 3.99 | **0.0005** | 2.90 | 0.0128 |
| tb | 1.85 | 0.294 | -11.7 | **3.03E-09** | -10.3 | **2.34E-07** |
| te | -0.79 | 0.465 | 8.81 | **6.02E-13** | 7.11 | **6.25E-09** |
| v | -2.29 | 0.0047 | 3.16 | **0.0005** | 1.20 | 0.190 |
| vb | -1.30 | 0.168 | 1.32 | 0.204 | -0.36 | 0.741 |
| ve | -4.51 | 0.0024 | 7.64 | **6.8E-06** | 5.01 | 0.0031 |
| w | -5.45 | 0.0058 | -15.4 | **8.49E-12** | -12.5 | **4.25E-08** |
| wb | -4.97 | 0.030 | -16.5 | **1.46E-10** | -14.6 | **3.02E-08** |
| we | -9.42 | 0.041 | -15.4 | 0.0040 | -8.62 | 0.105 |
| y | 2.67 | 0.023 | -3.47 | 0.0083 | -0.93 | 0.478 |
| yb | 4.89 | 0.0012 | -4.77 | 0.0042 | -1.66 | 0.327 |
| ye | -0.97 | 0.624 | -1.52 | 0.497 | 0.25 | 0.912 |

**^a^**Results from single logistic regressions of fractional amino acid content against expression level (E), solubility score (S), and usability (E*S > 11). Slope and p-value are shown. Parameters with p-values below the Bonferroni-corrected threshold of 0.0007 are shown in bold. The nomenclature describing amino acid content is described in Table 1 in the main text.

.

**Table S2. *Single logistic regressions on compound sequence parameters.*^a^**

|  | **Expression** | | **Solubility score** | | **Usability** | |
| --- | --- | --- | --- | --- | --- | --- |
| **Parameter** | **Slope** | **P-value** | **Slope** | **P-value** | **Slope** | **P-value** |
| netcharge | -0.026 | **7.32E-34** | -0.015 | **8.58E-11** | -0.021 | **1.74E-17** |
| numcharge | 0.0018 | 0.0037 | -0.0007 | 0.327 | 0.0006 | 0.412 |
| absnetcharge | -0.00004 | 0.992 | 0.029 | **1.74E-17** | 0.022 | **1.05E-10** |
| fracnetcharge | -4.78 | **1.05E-30** | -2.86 | **5.65E-10** | -4.13 | **8.8E-17** |
| fracnumcharge | 2.75 | **1.08E-12** | 5.77 | **3.76E-39** | 6.36 | **5.81E-45** |
| fracabsnetcharge | -2.21 | **8.15E-05** | 6.56 | **4.92E-22** | 3.80 | **5.88E-09** |
| sce | 1.46 | **9.1E-12** | 1.62 | **1.7E-11** | 2.39 | **6.85E-23** |
| esce | 0.91 | **5.33E-08** | 0.61 | 0.0013 | 1.17 | **8.25E-10** |
| gravy | -0.62 | **3.55E-19** | -0.68 | **7.31E-18** | -0.93 | **2.04E-31** |
| length | 0.00007 | 0.660 | -0.0011 | **2.23E-09** | -0.0009 | **2.25E-06** |
| diso | -0.67 | **2.14E-06** | 0.41 | 0.0096 | 0.043 | 0.795 |
| pi | -0.16 | **1.20E-51** | -.090 | **7.43E-14** | -0.13 | **2.77E-27** |

**^a^**Results of single logistic regressions against expression level (E) and solubility score (S) for compound sequence parameters. Slope, standard error, Z score, and p-value are shown. P-values below the Bonferroni-corrected threshold of 0.0007 are shown in bold. The nomenclature describing the compound sequence parameters is described in Table 1 in the main text.

**Table S3. *Parameter coefficients in final predictive models.*^a^**

|  | **Usability** | | **Usability w/ rare codons** | | **Expression** | | **Solubility score** | |
| --- | --- | --- | --- | --- | --- | --- | --- | --- |
| **Parameter** | **Slope** | **P-value** | **Slope** | **P-value** | **Slope** | **P-value** | **Slope** | **P-value** |
| ab |  |  |  |  |  |  | -4.82 | 0.0012 |
| c | -8.50 | 2.14E-06 | -6.54 | 0.0005 |  |  | -13.73 | 5.03E-11 |
| e |  |  |  |  | 2.75 | 0.028 |  |  |
| fb | -3.88 | 0.0198 | -4.17 | 0.015 |  |  | -10.67 | 3.39E-08 |
| h |  |  |  |  | 12.71 | 2.74E-14 | 10.81 | 6.7E-07 |
| i |  |  |  |  |  |  | -5.70 | 0.0056 |
| ke |  |  |  |  | 6.05 | 1.36E-10 |  |  |
| l | -2.23 | 0.0308 |  |  |  |  | -10.38 | 3.64E-09 |
| mb |  |  |  |  | 7.89 | 0.00027 |  |  |
| nb |  |  |  |  |  |  | 15.60 | 0.0028 |
| ne |  |  |  |  |  |  | 12.64 | 1.45E-09 |
| p |  |  |  |  |  |  | 4.16 | 0.01 |
| q |  |  |  |  | 9.73 | 7.25E-15 |  |  |
| qe | 9.86 | 2.74E-11 | 8.44 | 1.44E-06 |  |  | 15.43 | 9.75E-16 |
| r | -9.82 | 1.18E-24 |  |  |  |  | -7.24 | 2.56E-12 |
| s | -4.33 | 0.0006 | -3.20 | 0.015 |  |  |  |  |
| te | 4.36 | 0.0026 | 5.13 | 0.00037 |  |  | 8.16 | 3.39E-08 |
| v |  |  |  |  |  |  | -8.21 | 1.19E-05 |
| w | -6.00 | 0.0226 |  |  |  |  |  |  |
| fracnumcharge | 9.65 | 6.6E-27 | 12.11 | 3.67E-24 | 3.70 | 4.31E-05 | 20.27 | 2.12E-37 |
| absnetcharge | 0.015 | 3.18E-05 | 0.011 | 0.0018 |  |  |  |  |
| fracabsnetcharge |  |  |  |  | -4.88 | 3.73E-14 | 4.01 | 1.44E-07 |
| netcharge |  |  |  |  | -0.025 | 5.19E-20 |  |  |
| gravy | -0.45 | 0.0037 | -0.78 | 1.44E-06 | -0.55 | 2.14E-06 | 1.72 | 3.01E-06 |
| sce |  |  | -4.13 | 1.1E-07 |  |  | -4.88 | 9.17E-11 |
| esce | -1.90 | 3.17E-10 |  |  | -1.40 | 7.42E-07 |  |  |
| diso | -1.73 | 1.72E-14 | -1.59 | 4.52E-12 | -1.73 | 3.39E-21 | -1.09 | 2.47E-07 |
| **Rare Codons** | | | | | | | | |
| rare r |  |  | -11.33 | 2.38E-15 |  |  |  |  |
| common r |  |  | -9.00 | 3.59E-13 |  |  |  |  |
| rare i |  |  | -13.75 | 9.8E-11 |  |  |  |  |
| common i |  |  | 8.74 | 8.92E-09 |  |  |  |  |
| rare p |  |  | -6.84 | 0.0093 |  |  |  |  |
| **Score Cut-points** | | | | | | | | |
| 0 to 1 |  |  |  |  | -0.682 |  | -2.095 |  |
| 1 to 2 |  |  |  |  | -0.548 |  | -1.728 |  |
| 2 to 3 |  |  |  |  | -0.233 |  | -1.201 |  |
| 3 to 4 |  |  |  |  | 0.375 |  | -0.532 |  |
| 4 to 5 |  |  |  |  | 1.0468 |  | 0.0410 |  |

**^a^**Variable coefficients and p-values for final predictors for usability, usability including rare codon effects, expression level (E), and solubility score (S). Amino acid variables are either for total fractional content (*e.g.*, “c” for total cys) or for fractional content of predicted buried or exposed residues (*e.g.*, “ab” for buried ala or “ke” for exposed lys). The nomenclature describing the other sequence parameters is described in Table 1 in the main text. The cut-points between the 6 category outcomes (scores 0-5) are indicated for the ordinal logistic models for E and S values. See the Methods section for description of the calculations of outcome probability in logistic models.

**Figure S1.**

**
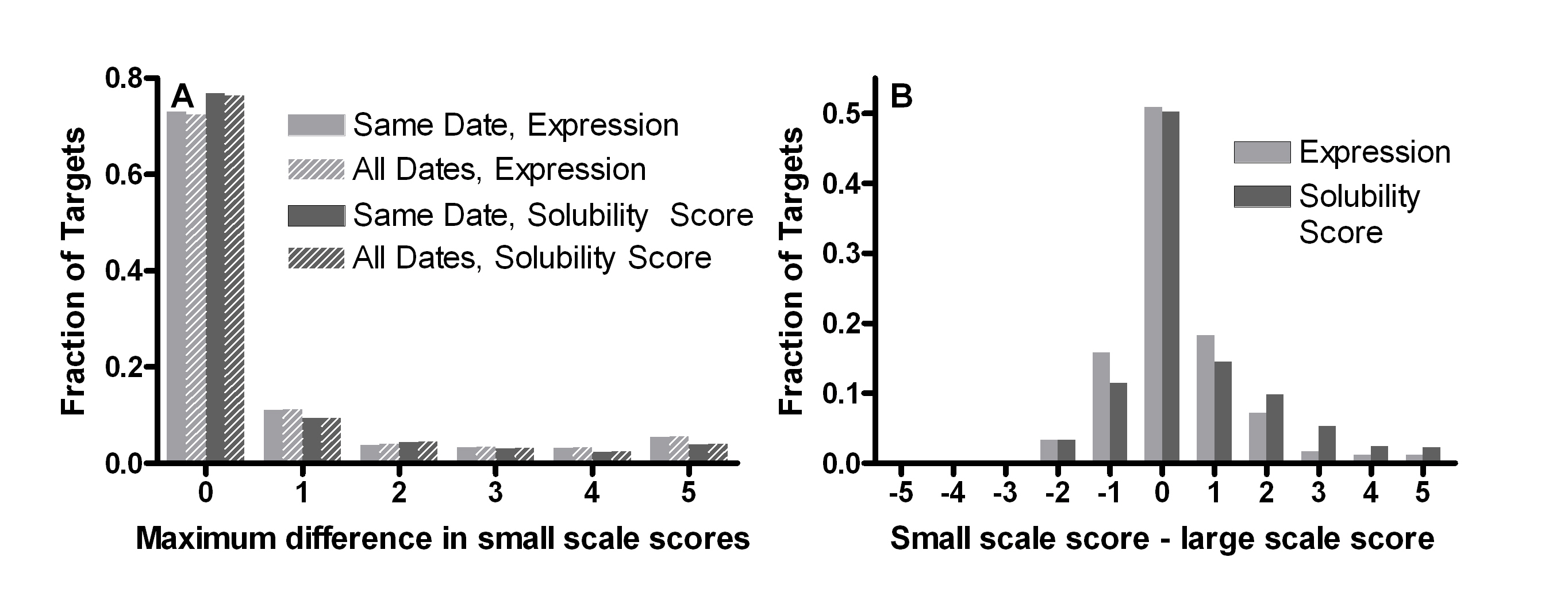
**

**Figure S1: *Consistency of expression and solubility scores across multiple measurements.*** Every protein construct evaluated in the NESG pipeline has at least two different clones (*i.e.,* transformants) scored for expression level (E) and solubility score (S) in small-scale robotic expression trials. “Usable” proteins with E*S > 11 generally undergo subsequent large-scale expression/purification procedures. **(A)** Histogram showing fractional distributions of the maximum difference between E or S scores from small-scale expression trials on different clones of the same protein construct evaluated on either the same day (solid bars, 38,188 observations from 18,660 protein constructs grouped into 18,857 comparison sets) or all days combined (striped bars, 38,278 observations from 18,660 protein constructs each in a single comparison set). Clones evaluated on different days derive from independent PCR-cloning procedures, while those evaluated on the same day generally derive from the same PCR-cloning procedure. Over 80% of all scores are within one unit of each other. **(B)** Histogram showing fractional distributions of the mathematical difference between E or S values measured in a small-scale expression experiment minus the corresponding values measured for the same clone in a subsequent large-scale expression experiment (9,408 observations for 4,607 clones from 4,487 protein constructs). Despite significant differences in culture volume and growth conditions, over 80% of clones give expression scores within one unit, and over 75% give solubility scores within one unit.

**Figure S2.**


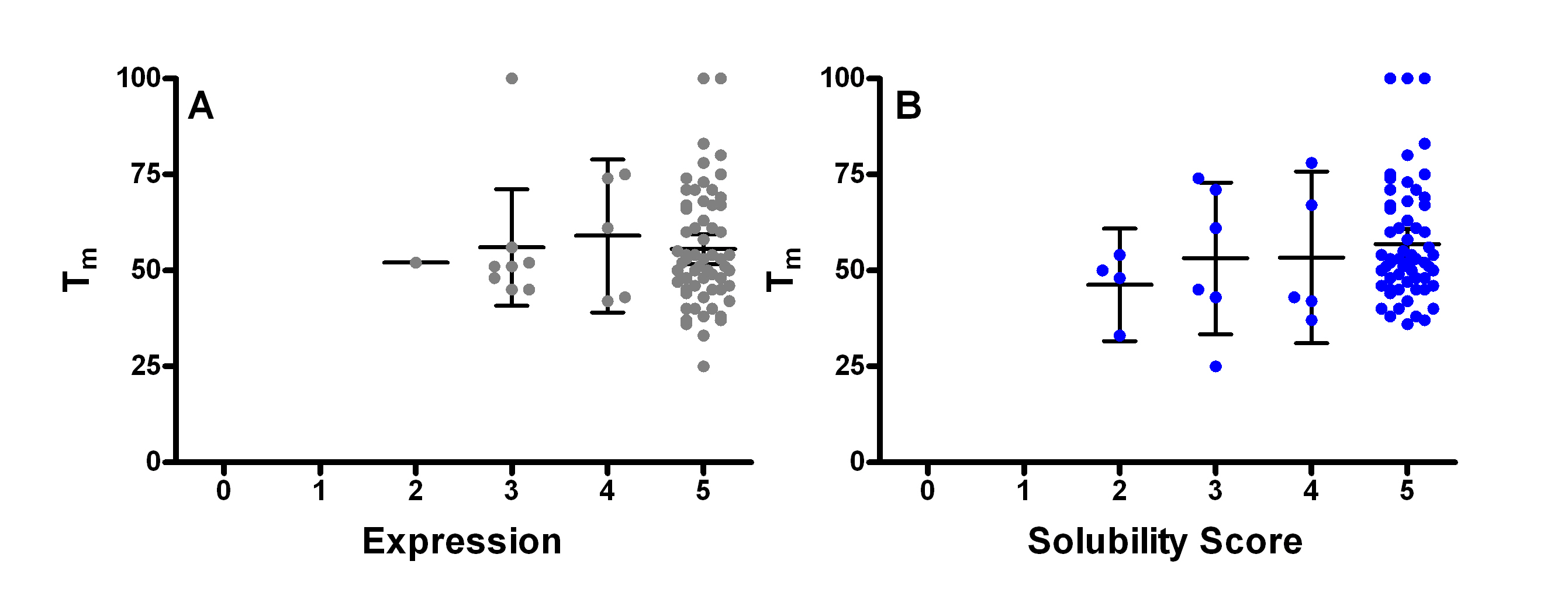
**Figure S2: *Relationship between protein stability and expression level or solubility score.*** Melting temperature (T_m_) was measured for 75 proteins in the Analysis Dataset using a hydrophobic-reporter dye. Results are binned according to their independently evaluated expression level (E: 0-5) or solubility score (S: 0-5). The mean T_m_ and 95% confidence interval are indicated for the proteins in each bin. Proteins with a product of expression level times solubility score (E*S) lower than 12 are not scaled-up for production by the NESG and therefore are not analyzed here. Among the proteins analyzed, there is no significant correlation between T_m_ and either E or S.

**Figure S3.**

**
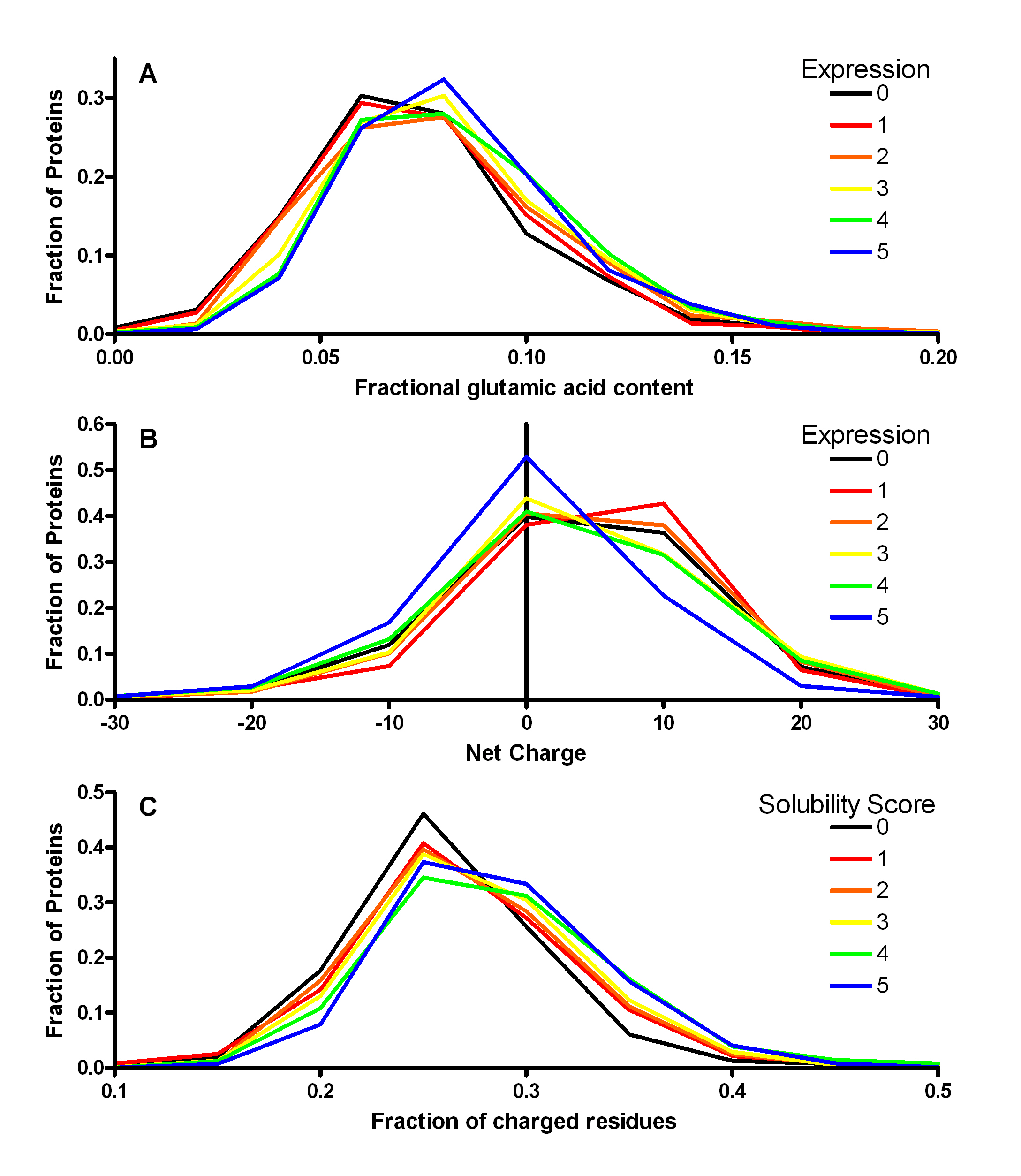
**

**Figure S3: *Sample score distributions.*** Proteins with different expression levels (E) and solubility scores (S) have significantly different distributions of sequence parameters. **(A,B)** Distributions of fractional Glu content (panel **A**, p = 5.08x10^-26^, N=7,733) and net charge (panel **B**, p = 7.32x10^-34^, N=7,733) are shown for proteins in the Analysis Dataset with each expression score from 0-5. **(C)**The distribution of the fraction of charged residues is shown for proteins with each solubility score (0-5) among proteins in the Analysis dataset with non-zero expression scores (p = 3.76x10^-39^, N=6,046).


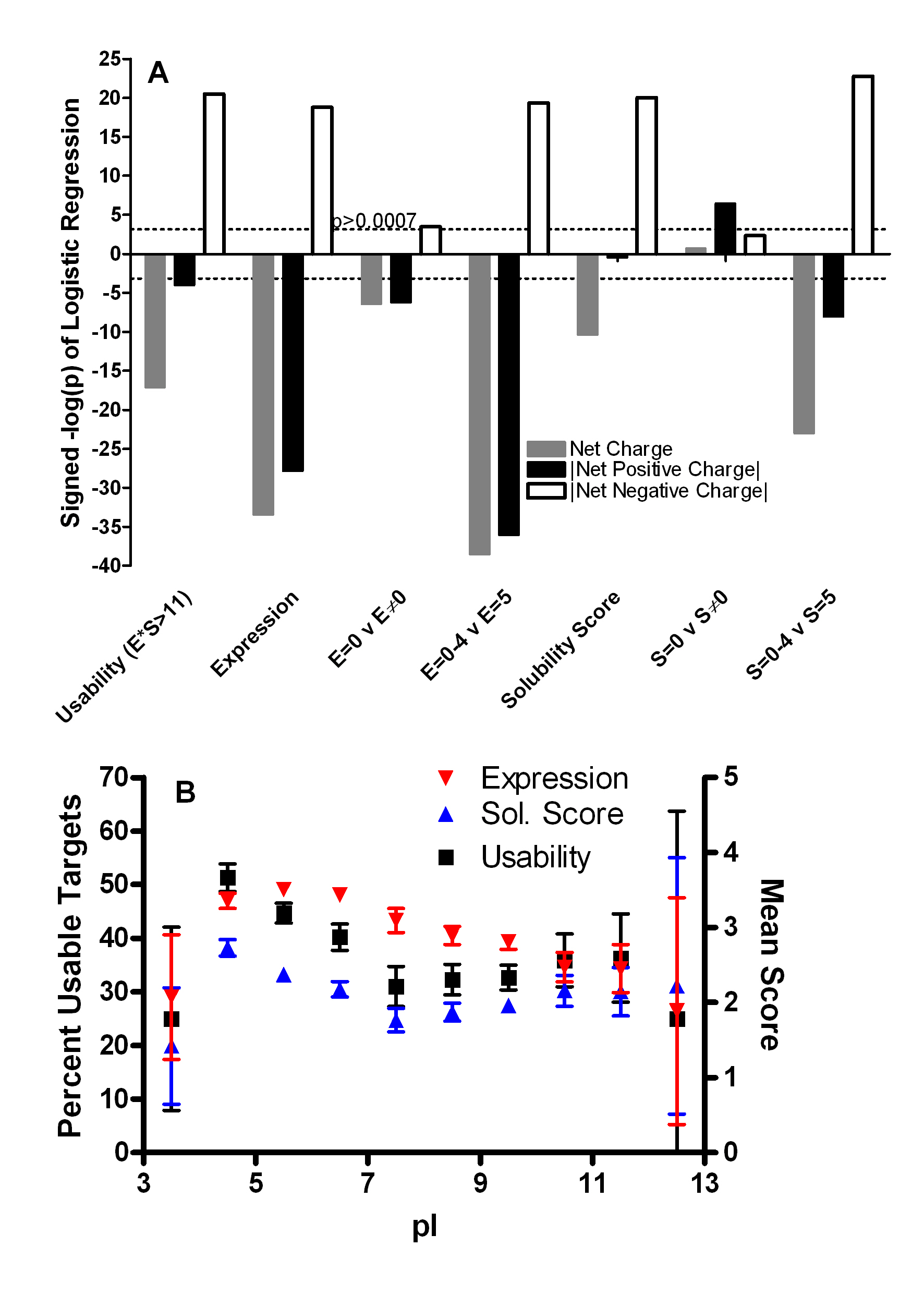
**Figure S4.**

**Figure S4: *Electrostatic charge and pI effects.* (A)** Comparison of influence of positively *vs.* negatively charged amino acids on experimental outcome. Because net electrostatic charge is a signed variable, its influence was dissected by separating it into two sub-variables: net positive charge, defined as net charge if net charge is positive and otherwise zero, and net negative charge, defined as the absolute value of net charge if net charge is negative and otherwise zero. All variables were divided by chain length to yield fractional variables. These variables were evaluated for their influence on the Analysis Dataset using single ordinal logistic regressions against expression level (E) or solubility score (S) and also using single binary logistic regressions against usability (*i.e.*, E*S>11) or permissiveness or enhancement for E or S (as defined in the main text and evaluated in Fig. S11 below). The bar graph shows the signed –log(p) values for these logistic regression, which accurately reflect the sign, magnitude, and significance for similarly distributed parameters. Net negative charge has a uniformly positive influence on E and S, while net positive charge has negative influence on E and a mixed influence on S. The negative influence of positive net charge is probably related in part to the effect of rare Arg codons in impeding translation; its influence on S becomes significantly positive (p = 0.00004) when regressed simultaneous with two variables representing separately the fractional content of Arg residues encoded by rare and common codons (data not shown). **(B)** The percent of usable proteins obtained and the mean E and S scores in bins of protein isoelectric point (pI). The error bars show 95% confidence limits based on counting statistics. Increasing pI has a non-monotonic but generally negative impact on expression level, solubility score, and usability. Modestly acidic proteins generally yield better results than neutral or basic proteins, consistent with the observed effects of net electrostatic charge.

**Figure S5.
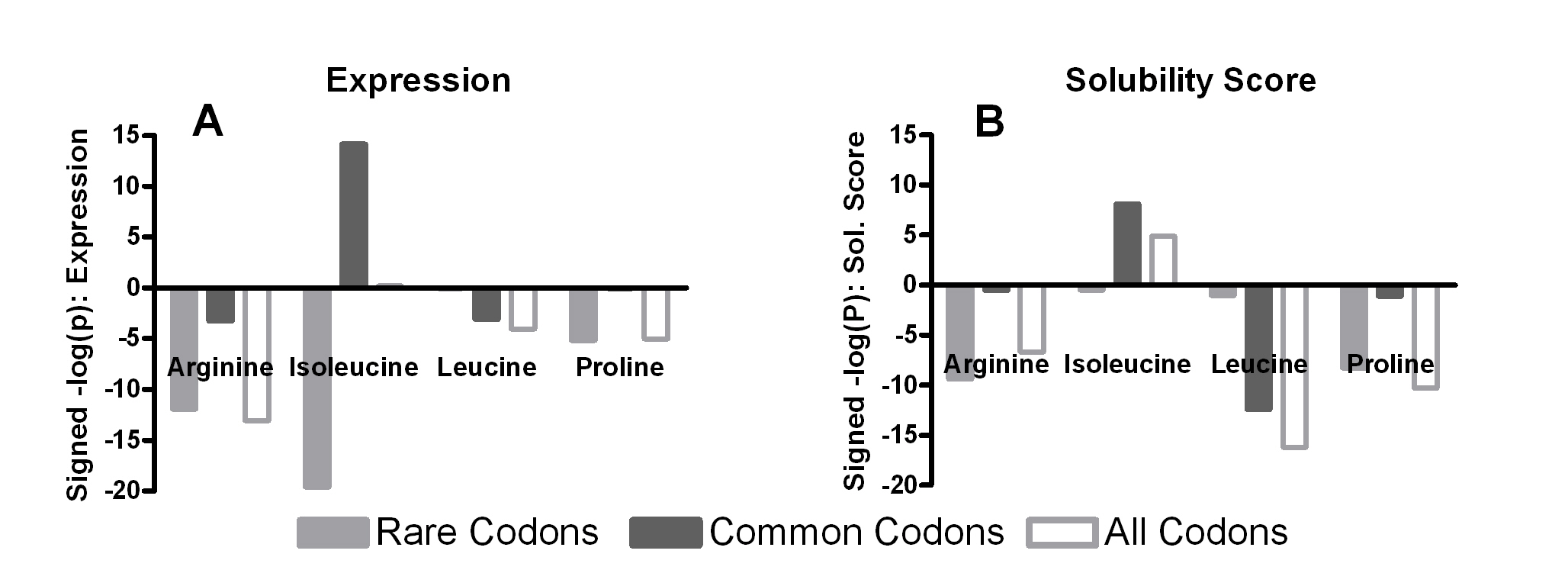
**

**Figure S5: *Influence of rare codons****.* Four amino acids are generally considered to be encoded in part by rare codons that are inefficiently translated: Arg, Ile, Leu, and Pro. For each of these amino acids, logistic regressions were performed on the Analysis Dataset to evaluate the influence on experimental outcome of the corresponding rare *vs.* common codons (*i.e.*, considered exclusively from one another). The ATA (Ile), CTA (Leu), CCC (Pro), and AGG, AGA, CGG, and CGA (Arg) codons were considered to be rare; except for CCC, each of these represents fewer than 8% of the codons for the corresponding amino acid found in the *E. coli* genome [87]. These two variables (*i.e.*, the fractional content of the protein encoded by the rare and common codons for the same amino acid) were analyzed in double ordinal logistic regressions for their correlation with expression level (panel **A**) or solubility score (panel **B**). Signed -log(p) values are shown for the results from these double regressions together with the result from the corresponding single regression for the total fraction of the corresponding amino acid (*i.e.*, the fraction of the protein encoded by the combination of the rare and common codons for that amino acid). The fraction of the protein encoded by rare Arg or Pro codons has a significant negative influence on both expression level and solubility score. In contrast, the fraction of the protein encoded by the rare Ile codon has a negative influence on expression level but not solubility score, providing further evidence that Ile is generally neutral or beneficial for protein solubility. The negative influence of Leu on expression level comes primarily from common codons, indicating that Leu residues themselves are generally deleterious for expression; given the extremely strong correlation between observed E and S values in the dataset, the influence of Leu on expression level could derive indirectly from its influence on solubility score.

**Figure S6**


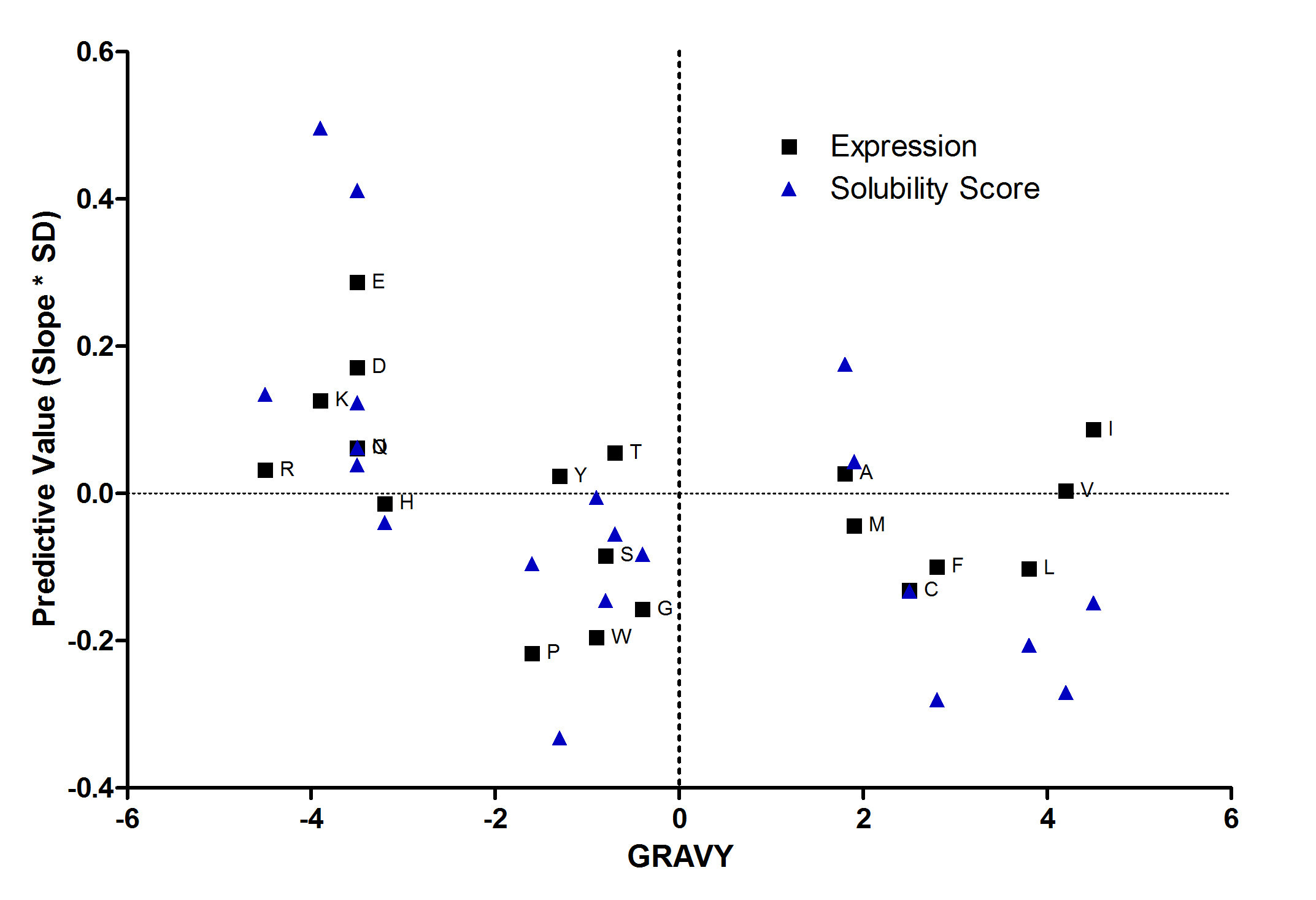


**Figure S6: *Predictive values for E and S in the Human Dataset vs. amino acid hydrophobicity***. The predictive values for either expression level or solubility score are plotted against hydrophobicity for single logistic regressions performed on the dataset comprising exclusively human proteins. Hydrophobicity shows a negative correlation with the predictive value for solubility in this dataset (r = 0.603, p = 0.0029), even when charged amino acids are excluded from consideration (r = 0.424, p = 0.049). The greater correlation with bulk amino-acid properties observed in this dataset could be attributable to a higher frequency of unfolded or misfolded proteins compared to the datasets dominated by bacterial proteins (Fig. 5 in the main text).


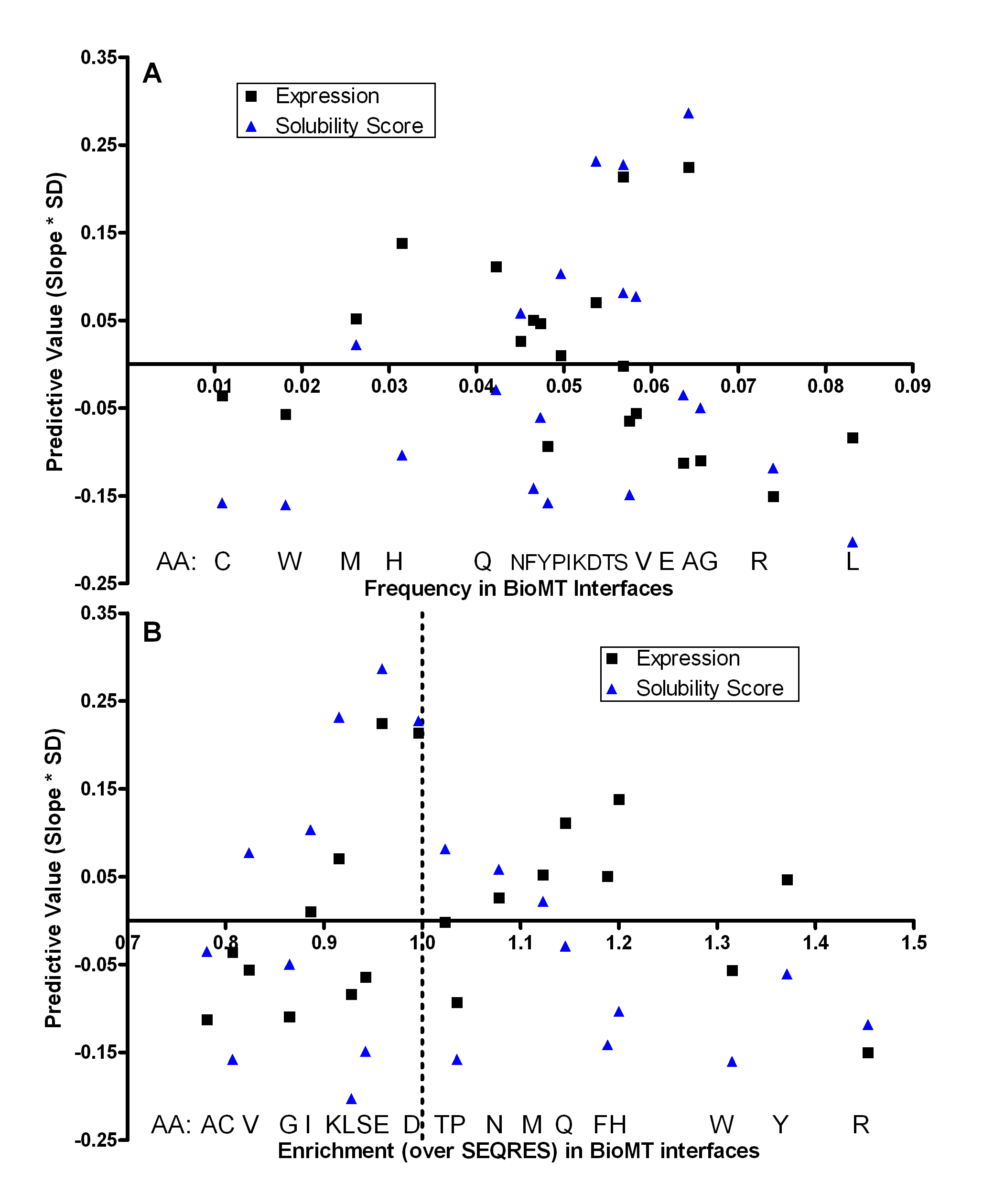
**Figure S7.**

**Figure S7: *Predictive values for E and S vs. participation in stable interprotein interfaces.*** To evaluate whether the observed influence of amino acid content on protein expression level or solubility score might be related to participation in stable interprotein interfaces, the predictive values for each amino acid in the Analysis Dataset were plotted against either the frequency of that amino acid in biological interfaces in the PDB as identified by the BioMT flag (panel **A**) or its enrichment in such interfaces (panel **B**), as defined by the ratio of the frequency of that residue in BioMT interfaces relative to its frequency in the SEQRES field of the same PDB depositions (V. Naumov and J.F. Hunt, manuscript in preparation). No significant correlation is observed with either metric in the complete dataset, although Leu which has the most deleterious influence on solubility is notably the most frequent amino acid in BioMT interfaces. (For frequency in BioMT interfaces, r_E_ = -0.20, p_E_ = 0.46, r_S_ = 0.19, and p_S_ = 0.49; for enrichment in BioMT interfaces, r_E_ = 0.08, p_E_ = 0.76, r_S_ = -0.289, and p_S_ = 0.319.)


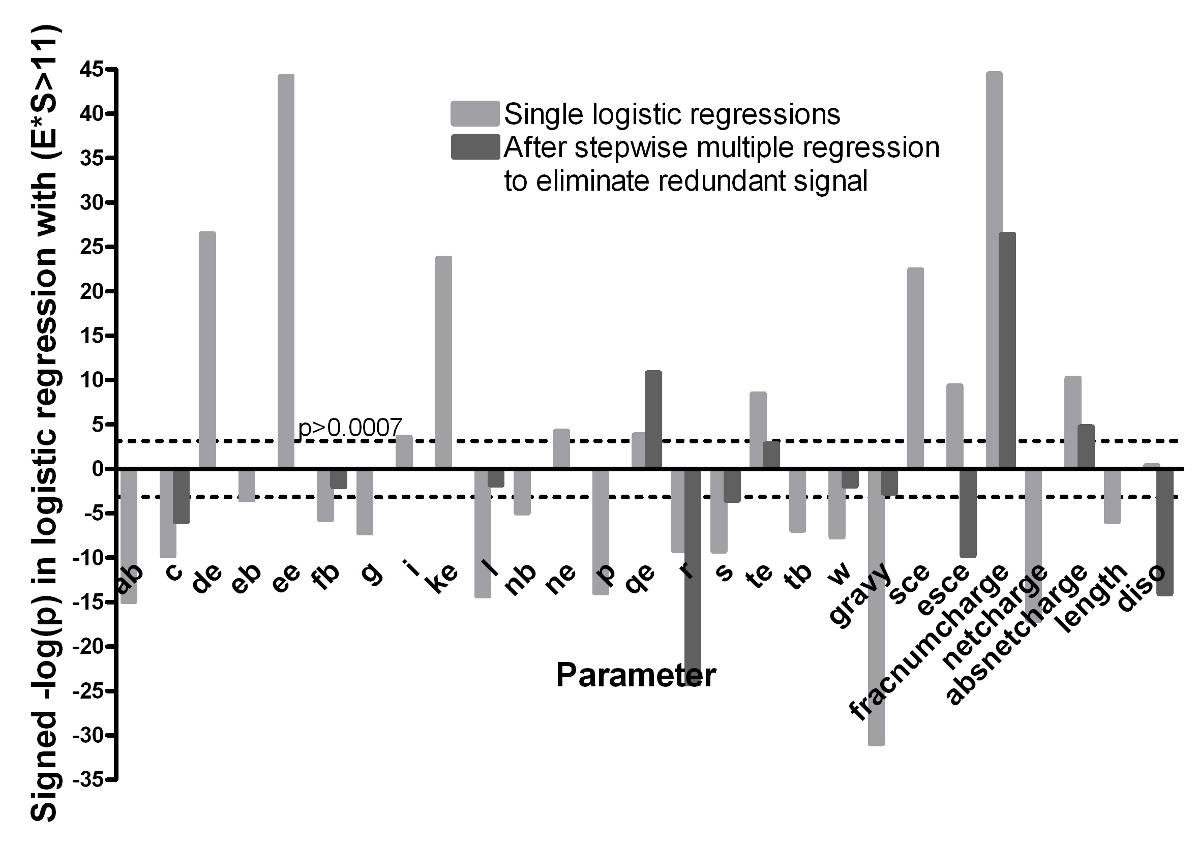
**Figure S8.**

**Figure S8: *Correlations between primary sequence parameters and usability.*** The Analysis Dataset was used to calculate logistic regressions between a large set of sequence parameters and practical protein usability, defined as having E*S > 11. Signed –log(p) values for parameters significant in individual regressions at the Bonferroni-corrected significance level (p < 0.0007) are shown in light gray. The parameters shown in dark gray remain significant after a stepwise Akaike Information Criterion multiple logistic regression to eliminate statistically redundant signals.


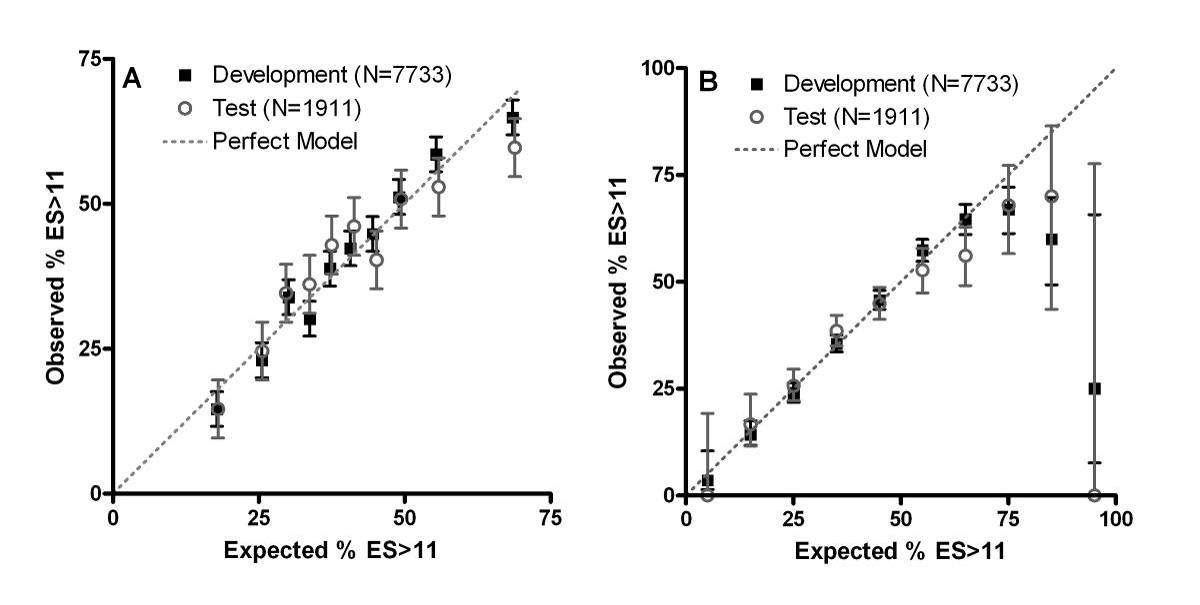
**Figure S9.**

**Figure S9: *Performance of a multiparameter predictor of usable protein yield including rare codon frequencies.***  For each of the four amino acids encoded in part by rare codons (Arg, Ile, Leu, and Pro), the total fractional amino-acid content was replaced with two variables separately quantifying the fractional content of that amino acid encoded by rare (as defined in the legend to Fig. S5) or common codons. Stepwise logistic regression was performed to create a final predictive model, as previously described for Fig. 3 in the main text. Performance on the Analysis Dataset is shown by filled squares, while performance on the corresponding Test Dataset is shown by open circles. **(A)** Model performance evaluated based on ten successive rank-ordered bins of equal protein population (773 proteins each for the development set and 191 each for the test set). The expected and observed fractions of usable proteins in each bin is plotted, with the error bars representing 95% confidence limits calculated based on counting statistics. **(B)** Model performance evaluated based on ten successive rank-ordered bins spanning equal intervals in yield of usable proteins (*i.e.*, E*S > 11). This predictive model describes both the Analysis Dataset (p = 9.2x10^-137^) and the Test Dataset (p = 3.3x10^-19^) somewhat better than the equivalent model not taking codon frequency into account (Fig. 8 in the main text).


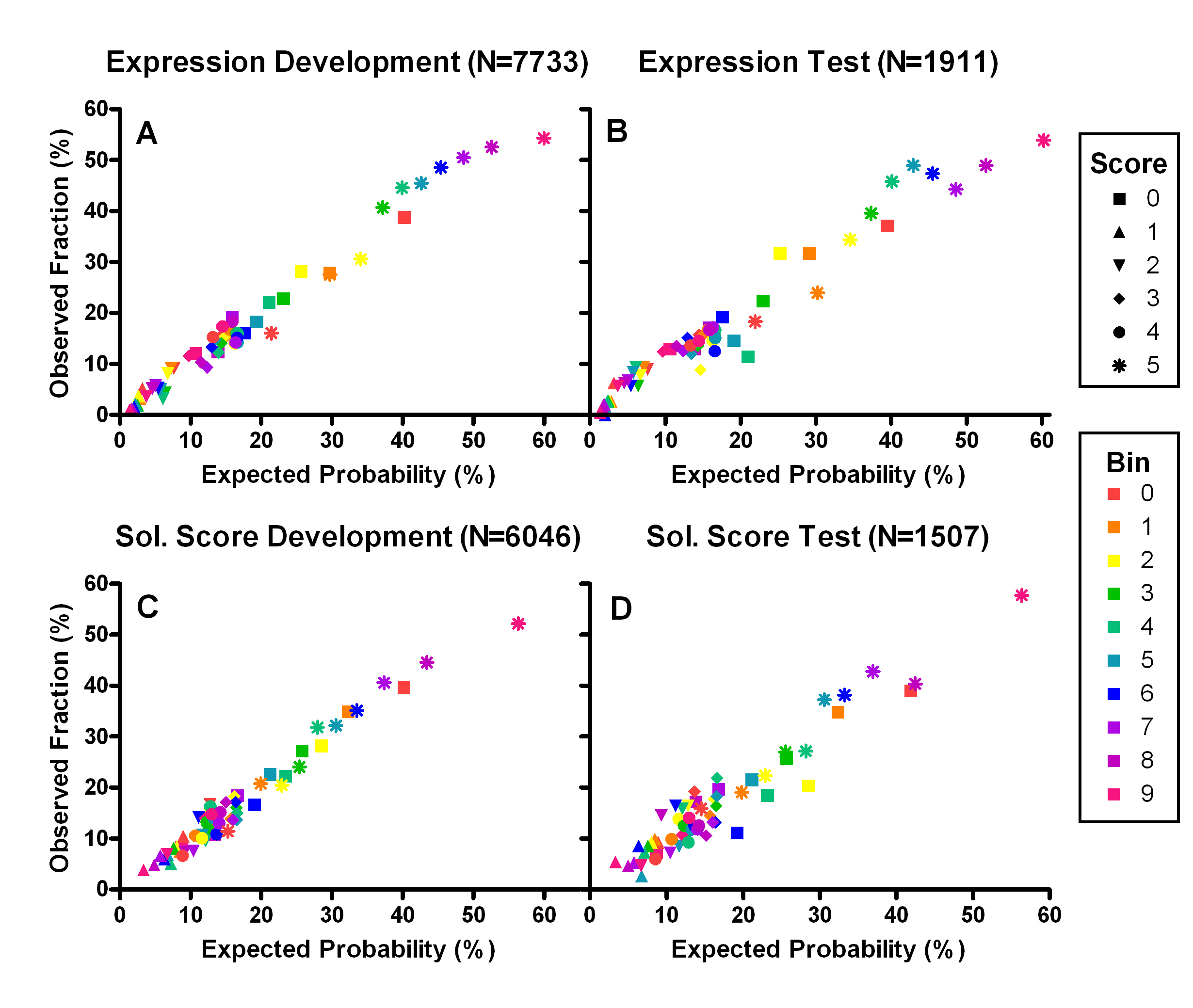
**Figure S10.**

**Figure S10: *Performance of multiparameter predictors of protein expression level and solubility score.*** Combined predictive metrics were developed as described for Fig. 3 in the main text. The output from an ordinal logistic regression is a set of probabilities for each outcome score rather than a single probability. Therefore, to evaluate reliability, the proteins were divided into 10 bins rank-ordered based on their logistic regression scores, with each containing an equal numbers of proteins (773 proteins per bin for the Analysis Dataset and 191 proteins per bin for the Test Dataset). The graph shows the expected *vs.* observed percentages of proteins at each score level in each such bin. Each of the 10 bins has 6 data points displayed in every panel, indicating the expected and observed percentage of proteins at each of score value from 0-5. Data from different rank-ordered bins are shown in different colors, ranging from red (low) through green (medium) to violet and pink (high), while data for the different predicted score values within each bin are represented by symbols of different shape, as indicated in the upper legend on the graph. For example, in expression bin number 9, 60% of proteins were expected to score 5 for expression, while 58% actually did. The predictive metrics successfully model both the Analysis Dataset (p_E_ = 4.9x10^-110^, p_S_ = 4.0x10^-109^) and the corresponding Test Dataset (p_E_ = 6.1x10^-17^, p_S_ = 7.4x10^-15^).


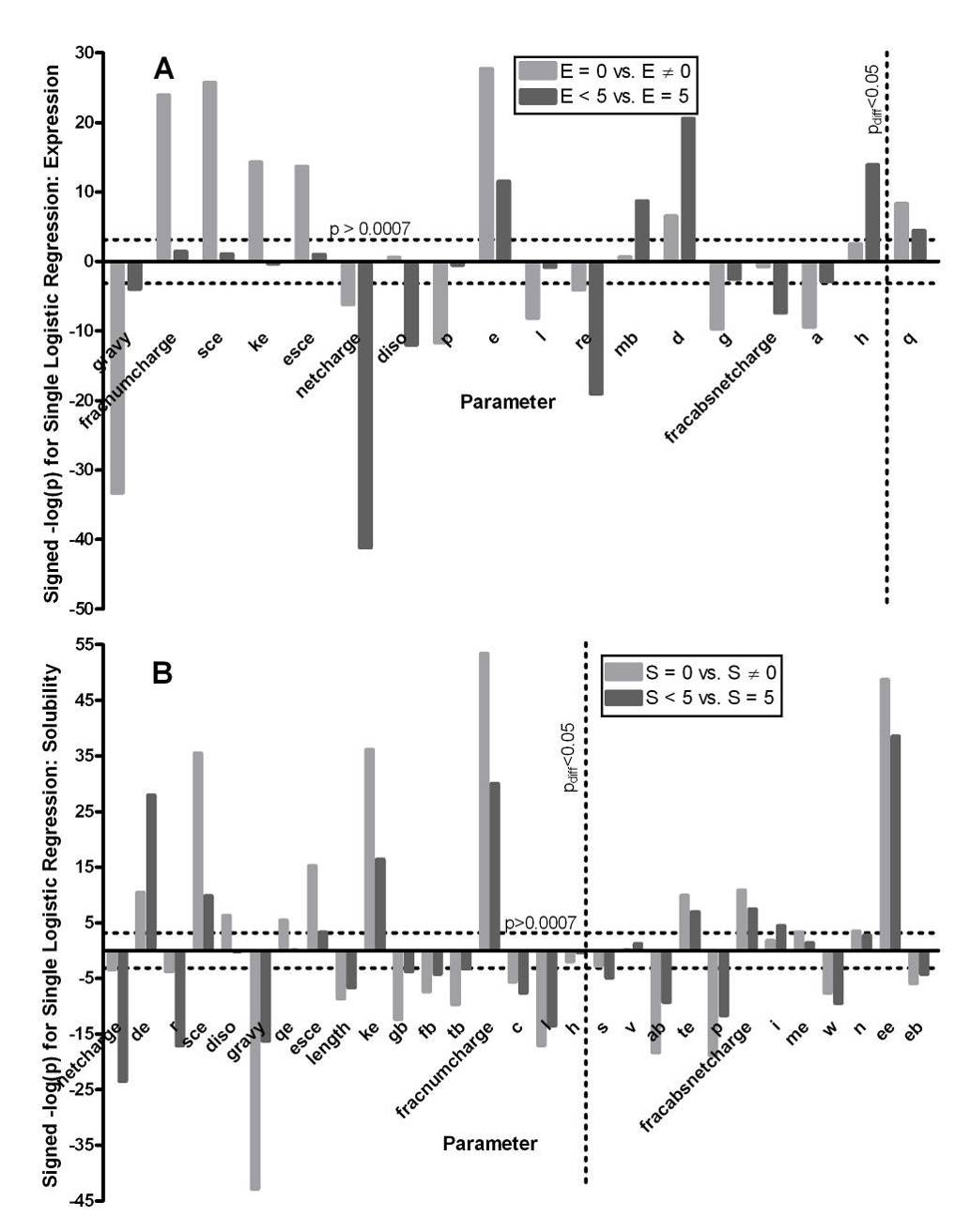
**Figure S11.**

**Figure S11: *Different parameter influence on permissiveness vs. enhancement.*** Some sequence parameters have significantly different influences throughout the observed range of expression and solubility scores. Permissive parameters are defined as influencing the probability of having a zero *vs.* non-zero score, while enhancing parameters are defined as influencing the probability of having the highest score *vs.* any lower score. Binary logistic regressions were used to identify sequence parameters significantly influencing the transition between these two score ranges (*i.e.*, 0 *vs.* 1-5 and 0-4 *vs.* 5) in the Analysis Dataset. In addition, a Brant test [83] was used to determine whether there are statistically significant differences in outcome ratio adjacent integer scores (*i.e.*, whether the ordinal regression model violated the assumption of equal odds increments between adjacent scores). These graphs show signed –log(p) values for each significantly predictive sequence parameter sorted by the significance of their Brant test. Dotted lines indicate statistical significance thresholds, of p < 0.05 for individual Brant statistics, and p < 0.0007 for Bonferroni-corrected single logistic regressions. Panel **A** shows regression results for expression level, while panel **B** shows regression results for solubility score.


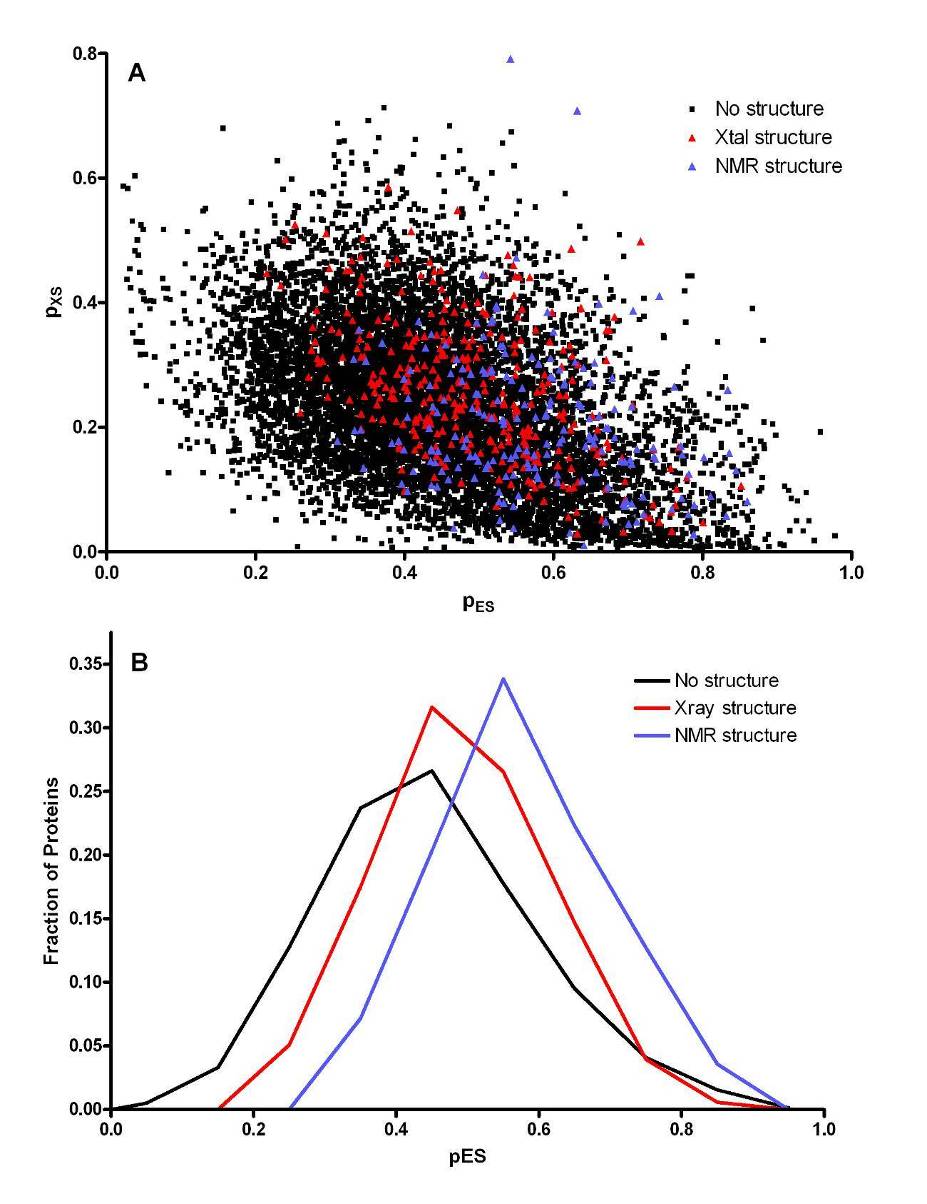
**Figure S12.**

**Figure S12: *The relationship between usability predictions and structure-determination rates.* (A)** Scatterplot showing the predicted probability of obtaining a usable protein preparation (P_XS_) *vs.* the predicted probability of obtaining an x-ray crystal structure (P_XS_) [5] for proteins in the Analysis Dataset segregated according to whether they yielded an x‑ray crystal structure (red triangles, n = 354), an NMR solution structure (blue triangles, n = 241), or no structure (black squares, n = 9,178). **(B)** Scaled histograms of the corresponding P_ES_ distributions. Based on unpaired heteroskedastic T-tests, the P_ES_ distributions are significantly different for proteins not giving a structure compared to those giving an x-ray crystal structure (p = 6.9x10^‑13^), for proteins not giving a structure compared to those giving an NMR solution structure (p = 6.9x10^-43^), and for proteins giving an x-ray crystal structure compared to those giving an NMR solution structure (p = 6.1x10^-15^).

**Figure S13.**

**
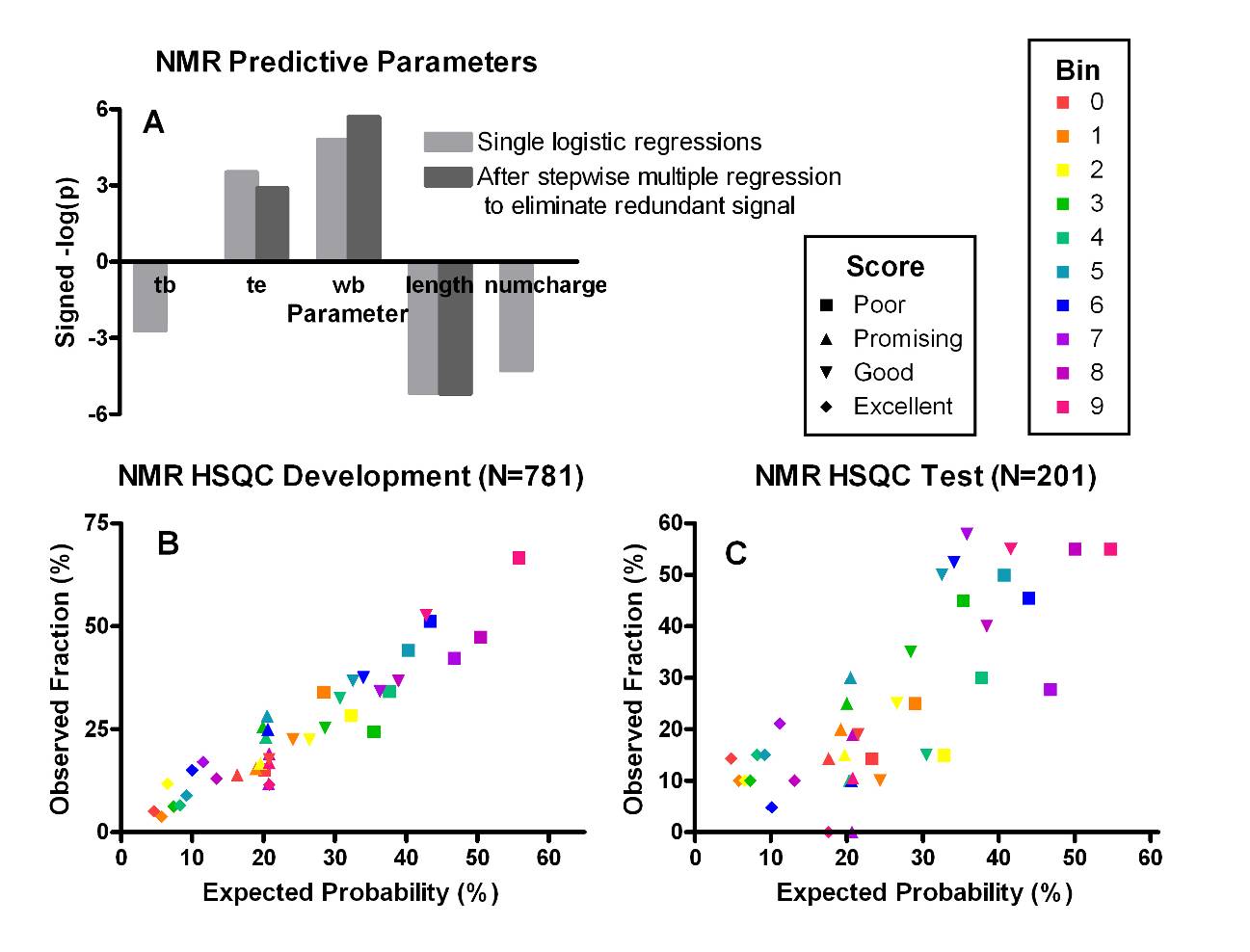
**

**Figure S13: *Correlations between sequence parameters and NMR HSQC screening score.*** Heteronuclear single quantum correlation (HSQC) NMR spectra were acquired from 982 expressed and soluble proteins in the NESG pipeline (*i.e.*, with E*S > 11). Spectra were scored as unfolded, poor, promising, good, or excellent based on visual inspection by expert spectroscopists. These categories were converted to numerical scores of 1 (poor) through 4 (excellent), and logistic regressions were used to evaluate potential correlations of these scores with same set of sequence parameters analyzed for correlations with expression and solubility. **(A)** Bar graph showing the negative of the log(p) values for the parameters remaining in the model after initial parameter culling and after stepwise logistic regression, as described in the Methods section. **(B**,**C)** Performance of the resulting predictive metric. Predicted and observed fractions of each integral score are shown for 10 bins of proteins rank-ordered according to their probability of yielding an NMR structure (*i.e.*, the same analysis shown in Fig. S10 for the metrics predicting E and S values). The NMR structure-determination metric predicts HSQC score significantly in the development dataset (panel **B**, n = 781, p = 1.5x10^-11^) but only marginally in the corresponding test dataset (panel **C**, n = 201, p = 0.07).


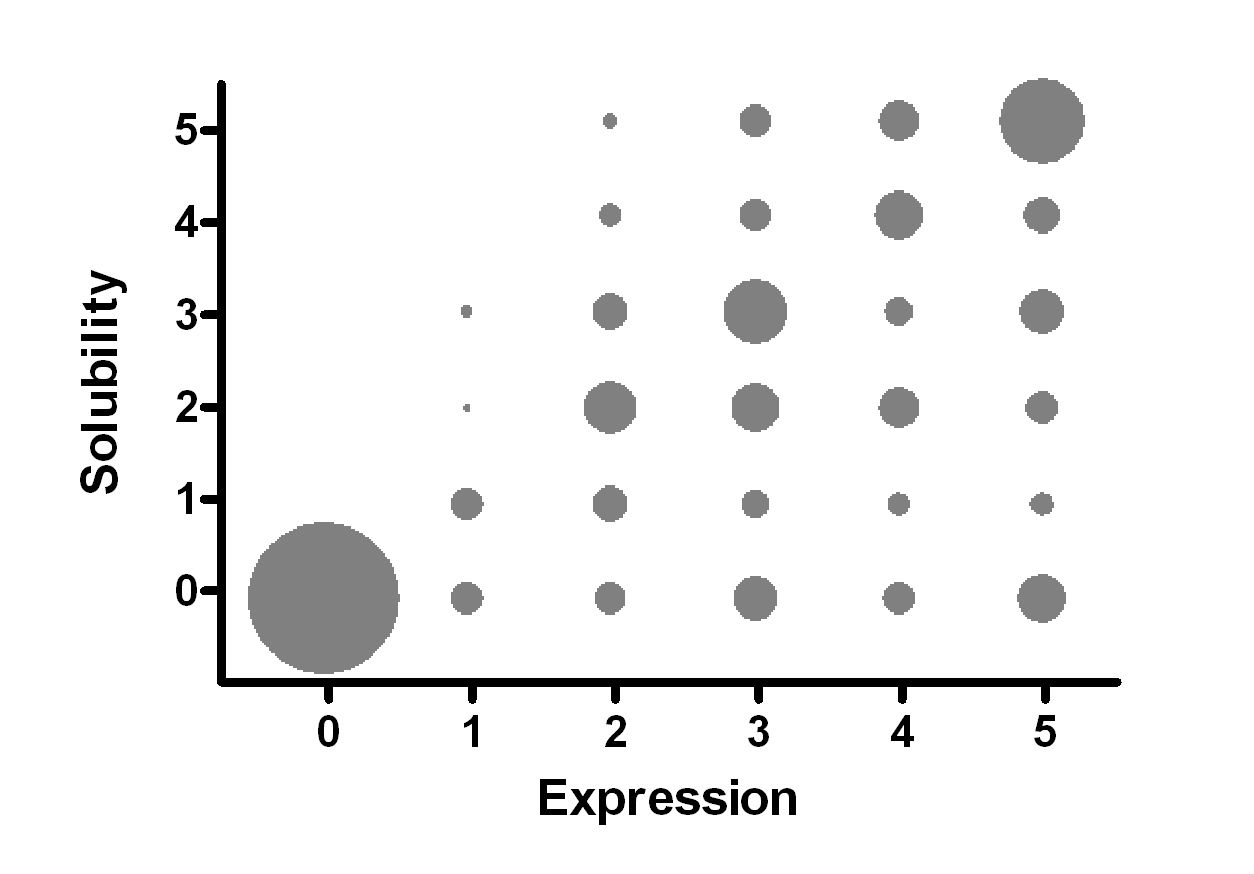
**Figure S14.**

**Figure S14: *Expression and solubility score distributions for potential signal-peptide-containing and transmembrane proteins.*** This bubble plot shows the E and S score distributions for the 865 proteins in the Analysis Dataset that have above-threshold predictions of having a signal peptide, lipopeptide, or transmembrane helix. (See Methods section in the main text for more information.) The presentation format is equivalent to that in Fig. 1C in the main text. The area of each point is proportional to the number of proteins with the indicated combination of expression and solubility scores.
